# GABA-A and NMDA receptor subunit mRNA expression is altered in the caudate but not the putamen of the postmortem brains of alcoholics

**DOI:** 10.3389/fncel.2014.00415

**Published:** 2014-12-05

**Authors:** Amol K. Bhandage, Zhe Jin, Igor Bazov, Olga Kononenko, Georgy Bakalkin, Esa R. Korpi, Bryndis Birnir

**Affiliations:** ^1^Molecular Physiology and Neuroscience, Biomedical Center, Uppsala UniversityUppsala, Sweden; ^2^Pharmacology, Institute of Biomedicine, University of HelsinkiHelsinki, Finland; ^3^Department of Pharmaceutical Bioscience (Biological Research on Drug Dependence), Biomedical Center, Uppsala UniversityUppsala, Sweden

**Keywords:** GABA, glutamate receptor, alcoholism, inhibition, excitation, AMPA, kainate

## Abstract

Chronic consumption of alcohol by humans has been shown to lead to impairment of executive and cognitive functions. Here, we have studied the mRNA expression of ion channel receptors for glutamate and GABA in the dorsal striatum of post-mortem brains from alcoholics (*n* = 29) and normal controls (*n* = 29), with the focus on the caudate nucleus that is associated with the frontal cortex executive functions and automatic thinking and on the putamen area that is linked to motor cortices and automatic movements. The results obtained by qPCR assay revealed significant changes in the expression of specific excitatory ionotropic glutamate and inhibitory GABA-A receptor subunit genes in the caudate but not the putamen. Thus, in the caudate we found reduced levels of mRNAs encoding the GluN2A glutamate receptor and the δ, ε, and ρ2 GABA-A receptor subunits, and increased levels of the mRNAs encoding GluD1, GluD2, and GABA-A γ1 subunits in the alcoholics as compared to controls. Interestingly in the controls, 11 glutamate and 5 GABA-A receptor genes were more prominently expressed in the caudate than the putamen (fold-increase varied from 1.24 to 2.91). Differences in gene expression patterns between the striatal regions may underlie differences in associated behavioral outputs. Our results suggest an altered balance between caudate-mediated voluntarily controlled and automatic behaviors in alcoholics, including diminished executive control on goal-directed alcohol-seeking behavior.

## Introduction

In the mammalian brain the areas identified as caudate and putamen are parts of the striatum and participate in neuronal circuits directing complex behavior (Postuma and Dagher, 2006; Grahn et al., 2008; Depoy et al., 2013). Evidence based on anatomical studies in animals and humans and functional imaging in humans show that the caudate participates in cognitive tasks whereas the putamen is the primary motor structure in the striatum (Alexander et al., 1986; Postuma and Dagher, 2006; Grahn et al., 2008). In humans there is a clear link between the caudate and the executive frontal areas whereas the putamen is strongly connected to the premotor and sensorimotor cortex (Postuma and Dagher, 2006; Grahn et al., 2008). In the normal mammalian brain, the caudate is thought to be involved in some aspects of automatic thinking whereas the putamen is more associated with automatic movements (Postuma and Dagher, 2006; Hardwick et al., 2013). Drugs of abuse are known to alter habits and complex behaviors that often involve activation of the striatum.

Glutamate and γ-aminobutyric acid (GABA) are the main excitatory and inhibitory neurotransmitters, respectively, in the brain and normally maintain the proper level of excitation in neuronal circuits (Smart and Paoletti, 2012). Both glutamate- and GABA-activated ionotropic receptors are multi-subunit channels (Smart and Paoletti, 2012). The ionotropic glutamate receptors are tetrameric ion channels, opened by glutamate and permeable to cations. They are subdivided into 4 families of receptors: AMPA (subunits GluA1-4), NMDA (subunits GluN1, GluN2A-D, GluN3A-B), kainate (subunits GluK1-5) and delta receptors (subunits GluD1-2) (Traynelis et al., 2010). The AMPA receptors normally mediate fast excitatory synaptic transmission and synaptic strength whereas the NMDA receptors, in addition to fast synaptic transmission, also regulate intracellular signaling and synaptic plasticity. The kainate receptors are present at both pre- and postsynaptic terminals and are thought to have a more modulatory role e.g., in regulating presynaptic transmitter release. The delta receptors to-date have no clear role in neurons (Smart and Paoletti, 2012). The GABA-A receptors are pentameric chloride-permeable anion channels that are opened by GABA. To-date, 19 different mammalian GABA-A receptor subunits have been identified: α1-6, β1-3, γ1-3, δ, ε, θ, π, and ρ1-3 (Olsen and Sieghart, 2009). Most neuronal GABA-A receptors are formed from 2α, 2β, and a 3rd type of subunits. The GABA-A receptors mediate both fast inhibitory synaptic transmission and the long-lasting tonic inhibition in neurons (Semyanov et al., 2004). It then depends on the genes that are transcribed which subtypes of the receptors are expressed in the neurons and this can change with developmental stage, brain region, type of neuron, synaptic activity and even diseases like alcoholism (Olsen and Sieghart, 2009).

Alcohol is the most common drug of abuse in Western societies. Chronic, excessive alcohol consumption may lead to changes in behavior, addiction and cognitive dysfunction (Jernigan et al., 1991; Harper, 1998; Harris et al., 2008; Spanagel, 2009). Acute exposure to ethanol modulates the ion channel function of both glutamate and GABA-A receptors. NMDA receptors are inhibited by ethanol in a concentration-dependent manner over a range of concentrations that produce intoxication (Lovinger et al., 1989; Gass and Olive, 2008). AMPA and kainate receptors are inhibited by high concentrations of alcohol (Gass and Olive, 2008; Moykkynen and Korpi, 2012) and ethanol inhibits several forms of neuronal plasticity, including long-term potentiation (LTP) (Blitzer et al., 1990; Morrisett and Swartzwelder, 1993; Givens and McMahon, 1995; Gass and Olive, 2008). In contrast to the effect on the glutamate transmission, ethanol, if anything, enhances the synaptic and tonic GABA transmission in neurons (Mihic et al., 1997; Sundstrom-Poromaa et al., 2002; Wallner et al., 2003; Wei et al., 2004; Borghese et al., 2006; Korpi et al., 2007; Baur et al., 2009). At low concentrations of ethanol (3–30 mM), tonic GABA-A receptor currents may be enhanced but as the alcohol concentration is raised (> 60 mM), GABA-A receptor-mediated synaptic transmission is also facilitated. In many brain regions, the main effect of ethanol on GABA signaling is enhancement of presynaptic release (Siggins et al., 2005). During chronic alcohol exposure, NMDA receptors are generally up-regulated and GABA-A receptors down-regulated in rodent models (Uusi-Oukari and Korpi, 2010; Lovinger and Roberto, 2013).

Alcohol-dependent disorders have been extensively studied in rodents but the human disorder is not fully mimicked by these models (Crabbe et al., 2013). A few studies have been conducted on post-mortem brain samples from alcoholics (Lewohl et al., 1997, 2001; Dodd and Lewohl, 1998; Buckley et al., 2000, 2006; Mayfield et al., 2002; Buckley and Dodd, 2004; Dodd et al., 2004; Flatscher-Bader et al., 2005, 2006; Kalsi et al., 2009; Jin et al., 2011a, 2014a,b; Domart et al., 2012). These studies are very valuable as they aid in the understanding of the mechanism underlying human alcohol dependence.

Here we have studied the changes that take place in the brains of alcoholics. We examined whether chronic alcohol consumption in humans changes the balance between excitation and inhibition in the post-mortem caudate and putamen by analyzing the mRNA expression levels of the glutamate- and GABA-activated receptor subunits. The results show significant changes in the expression of specific excitatory ionotropic glutamate receptor genes and specific inhibitory GABA-A receptor genes in the caudate but not the putamen of the striatum, indicating that alcoholism-related alterations in the expression of the main excitatory and inhibitory receptor subunits are not similar in adjacent brain regions.

## Materials and methods

### Human samples

Twenty nine human controls and 29 individuals with from chronic alcohol dependence were included in the study. All individuals were Caucasian males except one Asian in control group and one Australian in alcoholics group. The individuals suffering from alcoholism consumed ≥80 g alcohol per day during the majority of their adult lives, met the criteria for Diagnostic and Statistical Manual for Mental Disorders, fourth edition and National Health and Medical Research Council/World Health Organization and did not have liver cirrhosis, Wernicke–Korsakoff's syndrome, or multi-drug abuse history. Individuals in the control group had either abstained from alcohol completely or were social drinkers who consumed ≤20 g of alcohol per day on average. Individuals in the control group were matched to individuals suffering from alcoholism by age, post-mortem interval (PMI), brain pH, RNA quality indicator (RQI), and smoking history (Table S1). Post-mortem brain samples were from the rostral putamen and caudate nucleus. The samples from the caudate consisted mostly of the head with potentially some contribution from the anterior part of the body. The tissue was collected at the New South Wales Tissue Resource Center (TRC), University of Sydney, Australia (http://sydney.edu.au/medicine/pathology/trc/index.php). The description of the brain bank, the protocols, ethical approval and funding is described in Sheedy et al. (2008). All the samples from these two brain regions were collected from the same donors. All samples were collected by qualified pathologists under full ethical clearance and with informed, written consent from the next of kin. The detailed demographic data for all subjects are given in Table S2 in Supplementary Material.

### Total RNA isolation

Total RNA was isolated by using RNeasy Lipid Tissue Mini Kit (QIAGEN, Maryland, USA) and quantified with Nanodrop ND-1000 spectophotometer (Nanodrop Technologies, Inc.). The quality of RNA was evaluated by measuring RQI using Bio-Rad Experion (Bio-Rad Laboratories, Hercules, CA, USA) with Eukaryote Total RNA StdSens assay following the manufacturer's manual. RQI is equivalent to RNA integrity number (RIN) from Agilent (Denisov et al., 2008). RNA samples with RQI values greater than 5 are generally considered as suitable for RT-qPCR (Fleige and Pfaffl, 2006; Fleige et al., 2006). In this study, samples with RQI less than 5 were not used for experiments. Average RQI of the samples was 7.4 ± 0.2 (mean ± SEM; 84.5% samples have RQI equal or greater than 6 indicating high quality of isolated total RNA.

### Quantitative real-time RT-PCR

Total RNA (200 ng) was reverse transcribed into cDNA in a 20 μl reaction mixture using Superscript III reverse transcriptase (Invitrogen). RT negative control was performed by omitting reverse transcriptase in the reaction in order to confirm no genomic DNA contamination in the isolated RNA. Real-time PCRs were performed in a 10 μl reaction mixture containing 4 μl cDNA (1 ng), 1 × PCR reaction buffer, 3 mM MgCl_2_, 0.3 mM dNTP, 1 × ROX reference dye, 0.8 U JumpStart *Taq* DNA polymerase (Sigma-Aldrich), 5 × SYBR Green I (Invitrogen), and 0.4 μM each of forward and reverse primers. The gene-specific primer pairs (primer sequences shown in Table S3 in Supplementary Material) were designed using database GETPrime, EPFL (Gubelmann et al., 2011) or Primer Express Software version 3.0 (Applied Biosystems) and then validated using hippocampal cDNA from human brain by identification of a single peak in the melting curve and a single band with the expected size on an agarose gel. All samples were run in duplicate and the primers covered all available transcripts for that specific gene. Primer efficiency was not examined further as all PCR products were shorter than 200 base pairs. Amplification was performed in 384-well optical plates using the ABI PRISM 7900HT Sequence Detection System (Applied Biosystems) with an initial denaturation of 5 min at 95°C, followed by 45 cycles of 95°C for 15 s, 60°C for 30 s, and 72°C for 30 s. A melting curve was determined at the end of cycling to ensure the amplification of a single PCR product. Cycle threshold values (Ct) were determined with the SDS 2.4 and RQ Manager 1.2 softwares supplied with the instrument. The expression of each target gene relative to a normalization factor was calculated with Data Assist v2.0 using the 2^−Δ*Ct*^ method as previously described (Schmittgen and Livak, 2008). Reference gene beta actin (*ACTB*) for putamen (expression stability value *M* = 0.35) and caudate (expression stability value *M* = 0.42) was selected for normalization according to previously developed approach for analysis of reference genes (Johansson et al., 2007; Kuzmin et al., 2009; Bazov et al., 2011). As the expression of reference gene may vary between different brain regions of human alcoholics, it is of great importance to use validated stable reference gene for normalization.

### Statistical analysis

Statistical analysis was carried out using Statistica 11 and data were plotted with GraphPad Prism 6. Data were presented in scatter plots with 95% confidence interval or as box and whisker plots using the Tukey method to determine outliers (data points above or below the whiskers). Statistical analysis was then performed where the outliers were not included when data were compared. Normality of data distribution was analyzed using Shapiro–Wilk normality test (see Table S4 in Supplementary Material). The differences between groups were assessed by One-Way ANOVA with Bonferroni *post-hoc* test (normally distributed data) or nonparametric Kruskal–Wallis ANOVA on ranks with Dunn's *post-hoc* test (not normally distributed data). A general stepwise linear regression model was used to identify covariates (age, PMI, RQI, brain pH, smoking history). Variables with a significant association with group (controls and alcoholics) were included in the final statistical model as covariates. A significant level was set to *p* < 0.05.

## Results

The demographic characteristics of individuals in this study are shown in Table S2. There was no significant difference in age, PMI, brain pH, RQI, and proportions of smokers and non-smokers between individuals with or without alcohol dependence (Table S1). Expression of the four AMPA (GluA1-4), five kainate (GluK1-5), seven NMDA (GluN1, 2A-D, 3A-B), two delta (GluD1-2), and nineteen GABA-A (α1–6, β 1–3, γ1–3, δ, ε, θ, π, ρ 1–3) receptor subunit mRNAs were quantified by RT-qPCR in the samples collected from the caudate and putamen. The primers designed for GABA-A and glutamate receptor subunits covered all the transcripts available today for the particular subunit (Table S3).

### Decreased expression of GluN2A but increased expression of GluD1 and GluD2 in the caudate nucleus of alcoholics

**Figure 1:**
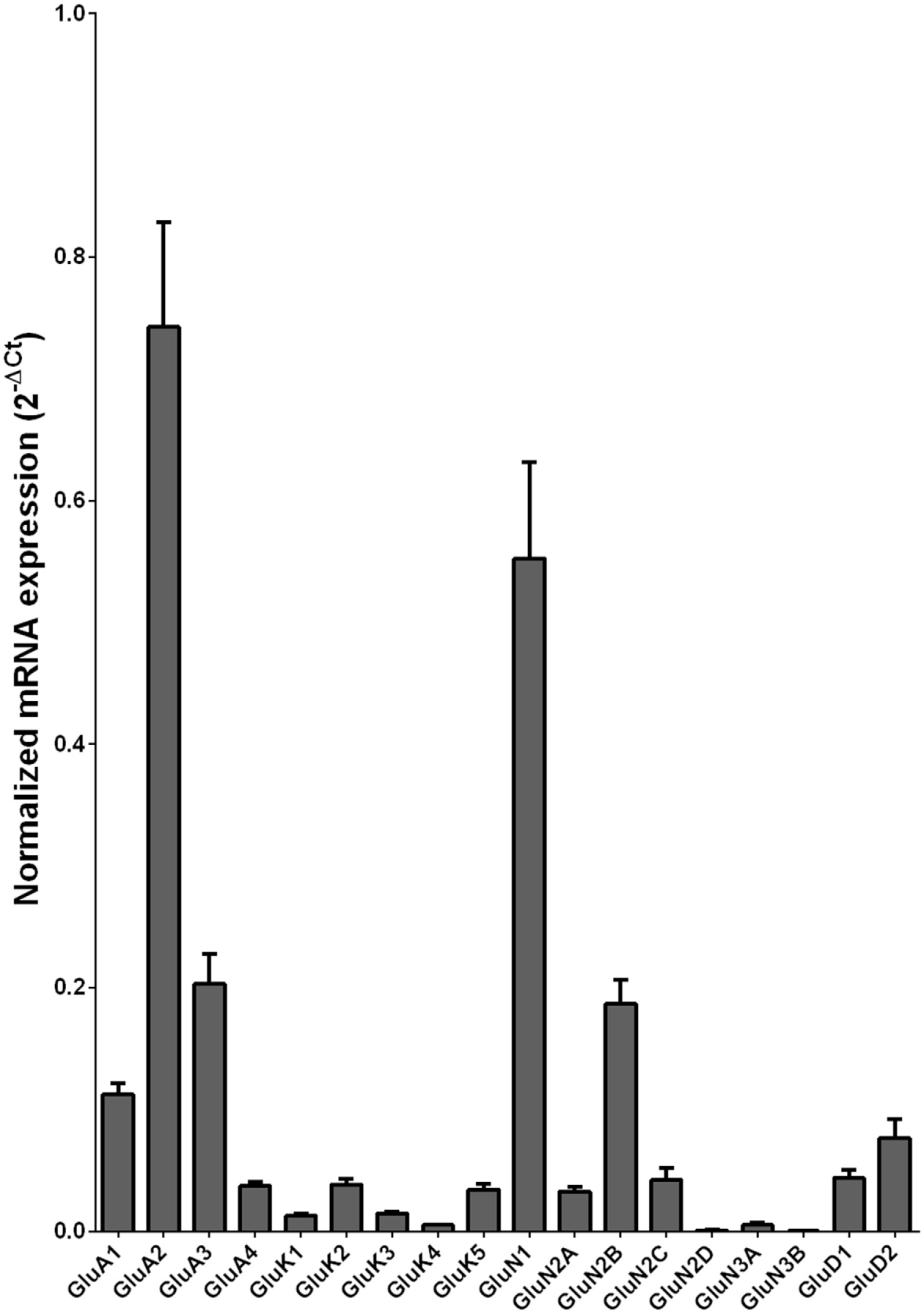
**Expression of ionotropic glutamate receptor subunit mRNAs in the caudate of control subjects (*n* = 29)**. The mRNA level of each subunit was normalized to reference gene *ACTB* and presented as mean ± SEM.

We examined the mRNA expression of the glutamate receptor subunits in the caudate from alcoholics and individuals without alcohol dependence. Normalized average expression levels of the different glutamate receptor subunits in the caudate of individuals without alcohol dependence are shown in Figure 1. Qualitatively, the high and medium levels of expression were defined as equal to or greater than that of GluN1 and GluA1, respectively. The results show high expression of GluA2 and GluN1, medium expression of GluA1, GluA3, and GluN2B, and low expression of other glutamate receptor subunit mRNAs.

**Figure 2:**
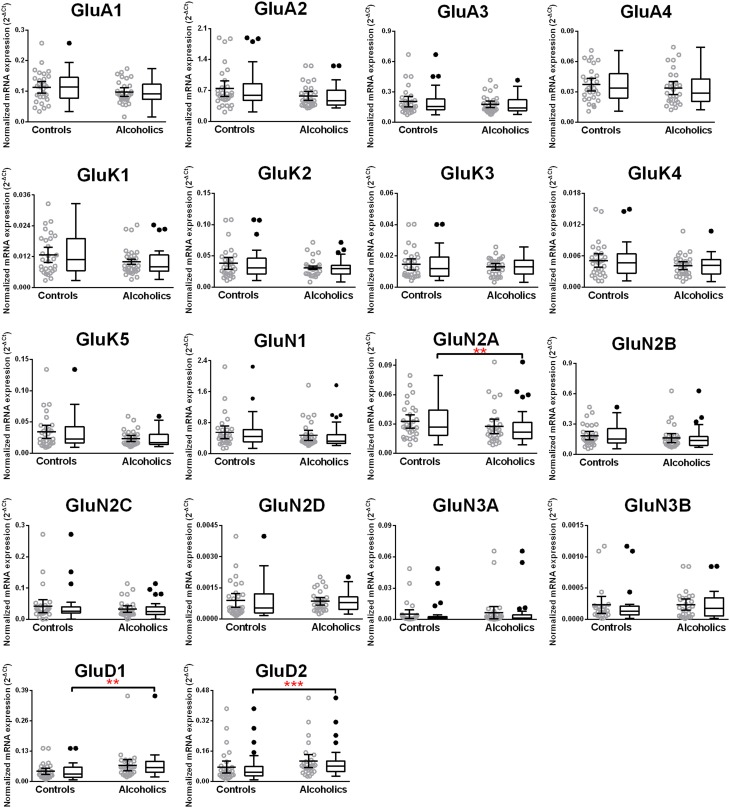
**Expression of ionotropic glutamate receptor subunits mRNA in the caudate of controls (*n* = 29) and alcoholics (*n* = 29)**. Data from each group is presented as scatter dot plot (°) with mean and 95% confidence interval and box and whiskers plot with median and whiskers plotted by Tukey method to determine outliers (•, above or below the whiskers). Statistical analysis was performed by excluding outliers. One Way ANOVA with Bonferroni *post-hoc* test, GluA1, *df* = 49, *p* = 0.40; GluK2, *df* = 49, *p* = 0.16; GluK4, *df* = 49, *p* = 0.14; GluN2C, *df* = 49, *p* = 0.38. Kruskal–Wallis ANOVA on ranks with Dunn's *post-hoc* test, GluA2, *H*_(1, 53)_ = 1.73, *p* = 0.19; GluA3, *H*_(1, 54)_ = 0.3, *p* = 0.86; GluA4, *H*_(1, 58)_ = 0.77, *p* = 0.38; GluK1, *H*_(1, 55)_ = 2.79, *p* = 0.095; GluK3, *H*_(1, 56)_ = 0.23, *p* = 0.63; GluK5, *H*_(1, 56)_ = 2.37, *p* = 0.12; GluN1, *H*_(1, 28)_ = 3.80, *p* = 0.051; **GluN2A**, *H*_(1, 54)_ = 6.82, *p* = 0.009; GluN2B, *H*_(1, 54)_ = 3.27, *p* = 0.07; GluN2D, *H*_(1, 56)_ = 1.68, *p* = 0.20; GluN3A, *H*_(1, 52)_ = 0.10, *p* = 0.75; **GluD1**, *H*_(1, 55)_ = 9.7, *p* = 0.0018; **GluD2**, *H*_(1, 50)_ = 10.75, *p* = 0.001. ^**^p < 0.01, ^***^p < 0.001.

We then examined the expression level between controls and alcoholics. The level of GluN2A mRNA was significantly decreased whereas the levels of GluD1 and GluD2 mRNAs were significantly increased in the alcoholics as compared to controls (Figure 2, Table 1). The significance between the two groups was not affected by age, brain pH, PMI, smoking history, or RQI. No individual in either group consistently expressed genes at higher level than the other individuals in the same group. In this study, box and whisker plots with median and whiskers were plotted using the Tukey method to determine outliers (data points above or below the whiskers). Statistical analysis was done where the outliers were not included when the data-sets were compared. The mRNA levels for the remaining subunits were not altered between the two groups in the caudate (Figure 2).

**Table 1:** **Fold change in expression of ionotropic glutamate and GABA-A receptor subunits**.

	**Caudate alcoholics/caudate controls**	**Caudate controls/putamen controls**
GluA1		1.32
GluA3		1.30
GluK5		1.75
GluN1		1.39
GluN2A	0.64	1.66
GluN2B		1.62
GluN2D		2.10
GluN3A		1.53
GluD1	1.61	1.75
GluD2	1.60	1.70
α1		2.91
α4		1.24
δ	0.74	
ε	0.50	
γ1	1.36	1.41
γ2		1.44
γ3		1.37
ρ2	0.29	

*Fold increase is calculated by this formula = Average 2^−Δ*Ct*^ of a group/Average 2^−Δ*Ct*^ of another group. Fold change in expression of ionotropic glutamate and GABA-A receptor subunits in alcoholics compared to non-alcoholic individuals in caudate; fold change in expression of ionotropic glutamate and GABA-A receptor subunits in caudate controls compared to putamen controls*.

### Decreased expression of the δ, ε, and ρ2 but increased expression of the γ1 GABA-A subunit mRNAs in the caudate nucleus of alcoholics

**Figure 3:**
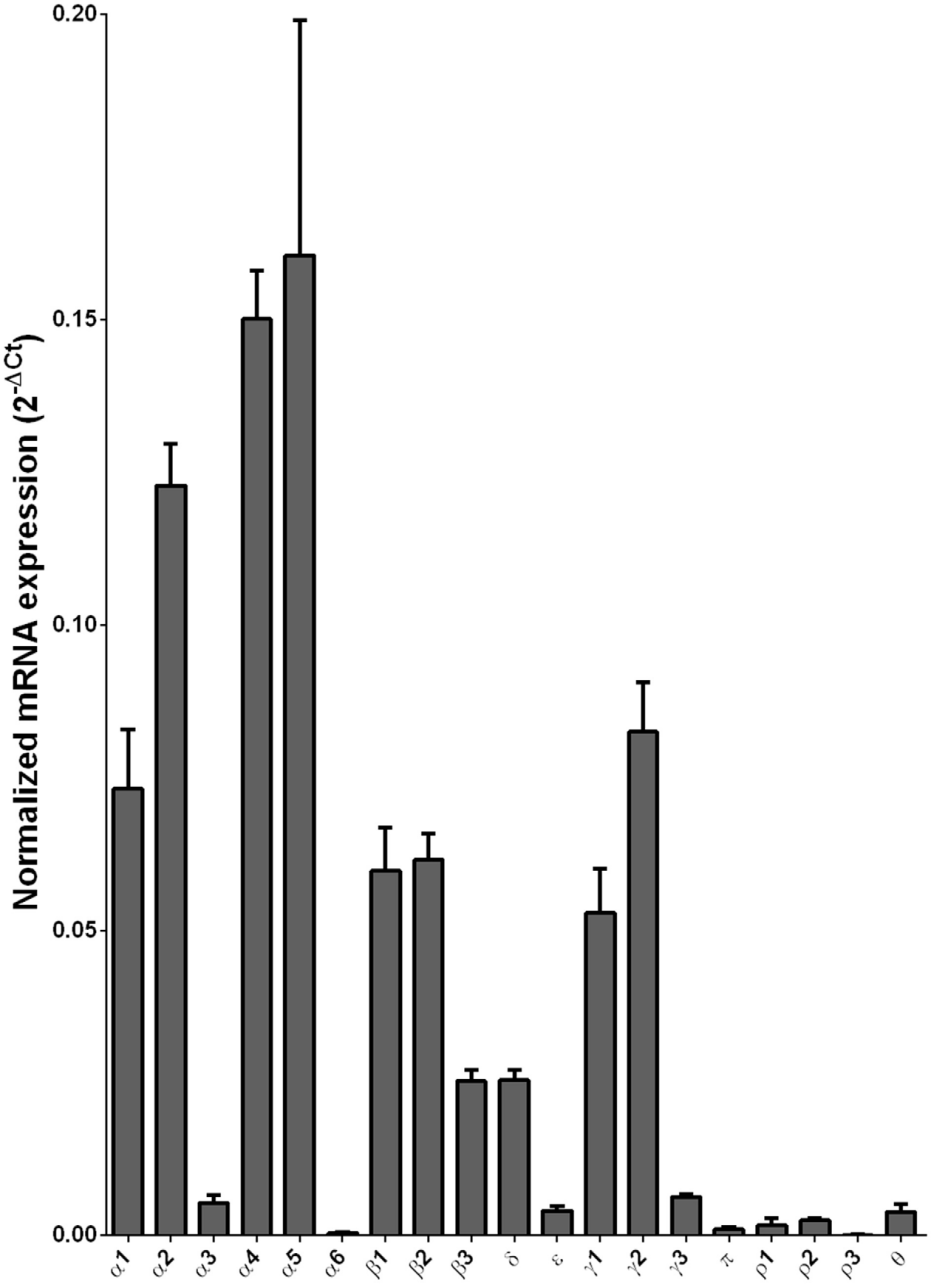
**Expression of GABA-A channel subunit mRNAs in the caudate of control subjects (*n* = 29)**. The mRNA level of each subunit was normalized to reference gene *ACTB* and presented as mean ± SEM.

We examined the mRNA expression of the GABA-A receptor subunits in the caudate from alcoholics and individuals without alcohol dependence. Normalized average expression levels of the different GABA-A receptor subunits in the caudate of individuals without alcohol dependence are shown in Figure 3. This qualitative expression of high and medium was defined as equal to or greater than that of γ1 and δ, respectively. The results show high expression of α1, α2, α4, α5, β1, β2, γ1, and γ2 but moderate expression of β3 and δ. The remaining subunits were expressed at a lower level.

**Figure 4:**
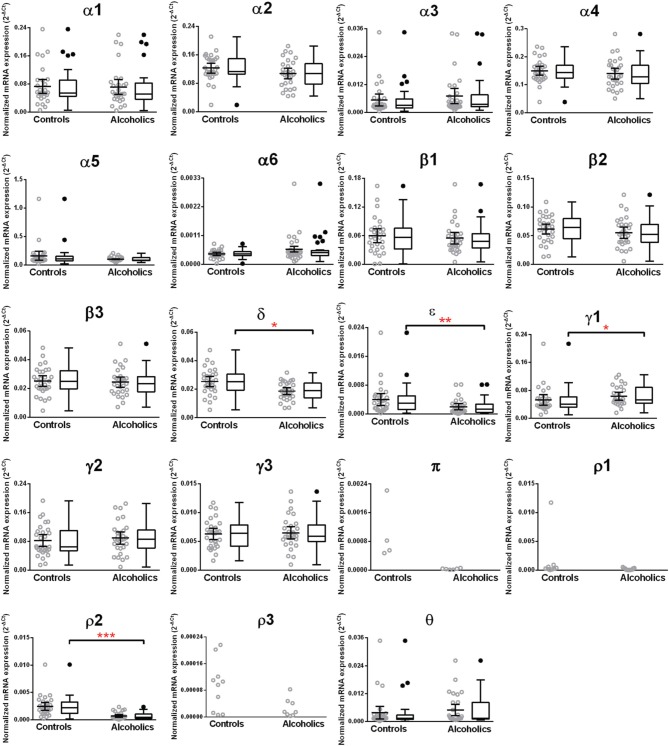
**Expression of GABA-A channel subunits mRNA in the caudate of controls (*n* = 29) and alcoholics (*n* = 29)**. Data from each group is presented as scatter dot plot (°) with mean and 95% confidence interval and box and whiskers plot with median and whiskers plotted by Tukey method to determine outliers (•, above or below the whiskers). Statistical analysis was performed by excluding outliers. One Way ANOVA with Bonferroni *post-hoc* test, α1, *df* = 47, *p* = 0.83; α2, *df* = 47, *p* = 0.17; α4, *df* = 47, *p* = 0.33; β 1, *df* = 47, *p* = 0.42; β 2, *df* = 47, *p* = 0.57; β 3, *df* = 47, *p* = 0.81; δ, *df* = 47, *p* = 0.021; γ2, *df* = 47, *p* = 0.22; γ3, *df* = 47, *p* = 0.79. Kruskal–Wallis ANOVA on ranks with Dunn's *post-hoc* test, α3, *H*_(1, 51)_ = 3.07, *p* = 0.08; α5, *H*_(1, 56)_ = 0.86, *p* = 0.35; α6, *H*_(1, 51)_ = 0.0072, *p* = 0.93; ε, *H*_(1, 53)_ = 8.41, *p* = 0.0037; **γ1**, *H*_(1, 53)_ = 5.94, *p* = 0.015; θ, *H*_(1, 54)_ = 1.59, *p* = 0.19; **ρ2**, *H*_(1, 55)_ = 23.68, *p* = 0.00001. ^*^p < 0.05, ^**^p < 0.01, ^***^p < 0.001.

The caudate levels of mRNAs encoding the δ, ϵ, and ρ2 subunits were significantly decreased whereas that of the γ1 subunit was increased in the alcoholics as compared to controls (Figure 4, Table 1). The mRNA levels for the remaining subunits were not altered between the two groups (Figure 4). The significance between the two groups was not affected by age, brain pH, PMI, smoking history, or RQI. No individual in either group consistently expressed genes at higher level than the other individuals in the same group.

### Unaltered mRNAs expression of glutamate and GABA-A receptor subunits in the putamen of alcoholics

**Figure 5:**
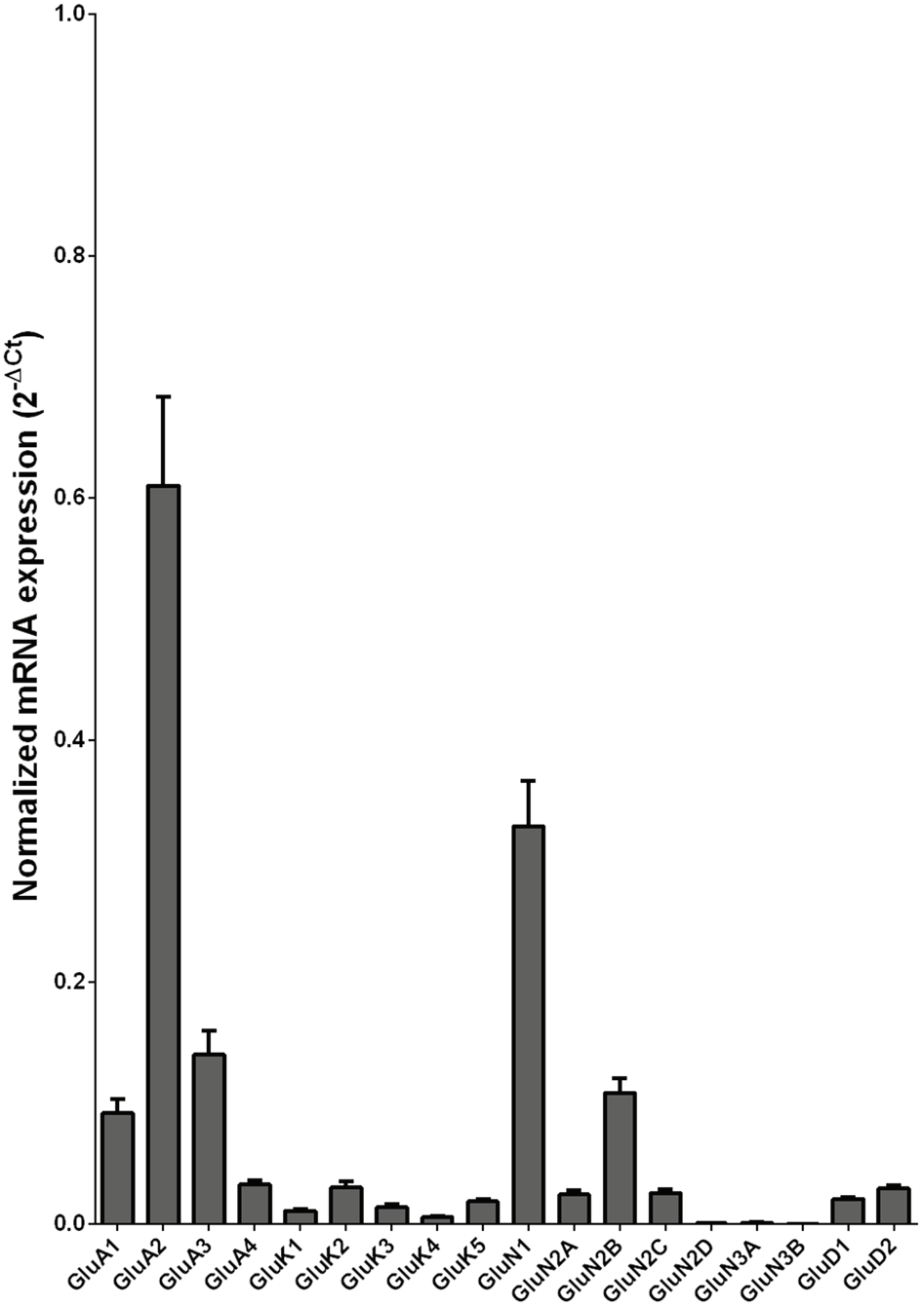
**Expression of ionotropic glutamate receptor subunit mRNAs in the putamen of control subjects (*n* = 29)**. The mRNA level of each subunit was normalized to reference gene *ACTB* and presented as mean ± SEM.

We examined the mRNA expression of the glutamate and GABA-A receptor subunits in the putamen from alcoholics and individuals without alcohol dependence. Normalized average expression levels of the different receptor subunits in the putamen of individuals without alcohol dependence are shown in Figures 5, 7. This qualitative expression of high and medium was defined as equal to or greater than that of GluN1 and GluA1, respectively, for glutamate receptors (Figure 5), and as equal to or greater than that of γ1 and δ, respectively, for the GABA-A receptors (Figure 7). For glutamate receptors, the results showed high expression of GluA2 and GluN1, medium expression of GluA1, GluA3, and GluN2B, but lower expression of other glutamate receptor subunit mRNAs. For GABA-A receptors, the results showed high expression of α2, α4, α5, β1, β2, γ1, and γ2 but moderate expression of α1, β3, and δ. The remaining GABA-A subunits were expressed at a lower level.

**Figure 6:**
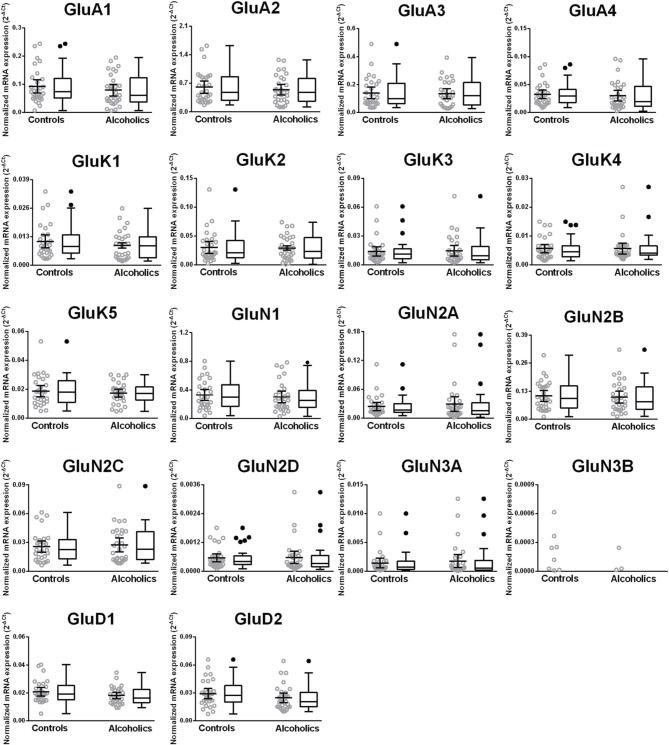
**Expression of ionotropic glutamate receptor subunits mRNA in the putamen of controls (*n* = 29) and alcoholics (*n* = 29)**. Data from each group is presented as scatter dot plot (°) with mean and 95% confidence interval and box and whiskers plot with median and whiskers plotted by Tukey method to determine outliers (•, above or below the whiskers). Statistical analysis was performed by excluding outliers. Kruskal–Wallis ANOVA on ranks with Dunn's *post-hoc* test, GluA1, *H*_(1, 56)_ = 0.34, *p* = 0.56; GluA2, *H*_(1, 58)_ = 0.2, *p* = 0.66; GluA3, *H*_(1, 57)_ = 0.03, *p* = 0.86; GluA4, *H*_(1, 56)_ = 0.69, *p* = 0.41; GluK1, *H*_(1, 56)_ = 0.38, *p* = 0.54; GluK2, *H*_(1, 57)_ = 0.16, *p* = 0.69; GluK3, *H*_(1, 54)_ = 0.0012, *p* = 0.97; GluK4, *H*_(1, 53)_ = 0.0079, *p* = 0.93; GluK5, *H*_(1, 57)_ = 0.0041, *p* = 0.95; GluN1, *H*_(1, 57)_ = 0.66, *p* = 0.42; GluN2A, *H*_(1, 53)_ = 1.22, *p* = 0.27; GluN2B, *H*_(1, 57)_ = 0.47, *p* = 0.49; GluN2C, *H*_(1, 57)_ = 0.057, *p* = 0.81; GluN2D, *H*_(1, 50)_ = 0.28, *p* = 0.60; GluN3A, *H*_(1, 53)_ = 0.98, *p* = 0.32; GluD1, *H*_(1, 58)_ = 2.07 *p* = 0.15; GluD2, *H*_(1, 56)_ = 2.42, *p* = 0.12.

We then examined the expression levels between controls and alcoholics. No significant difference was detected in mRNAs expression levels for any of the glutamate subunits (Figure 6) nor for any GABA-A receptor subunits between the two groups (Figure 8) in the putamen. Age, brain pH, PMI, smoking history, or RQI did not alter the significance between the two groups.

**Figure 7:**
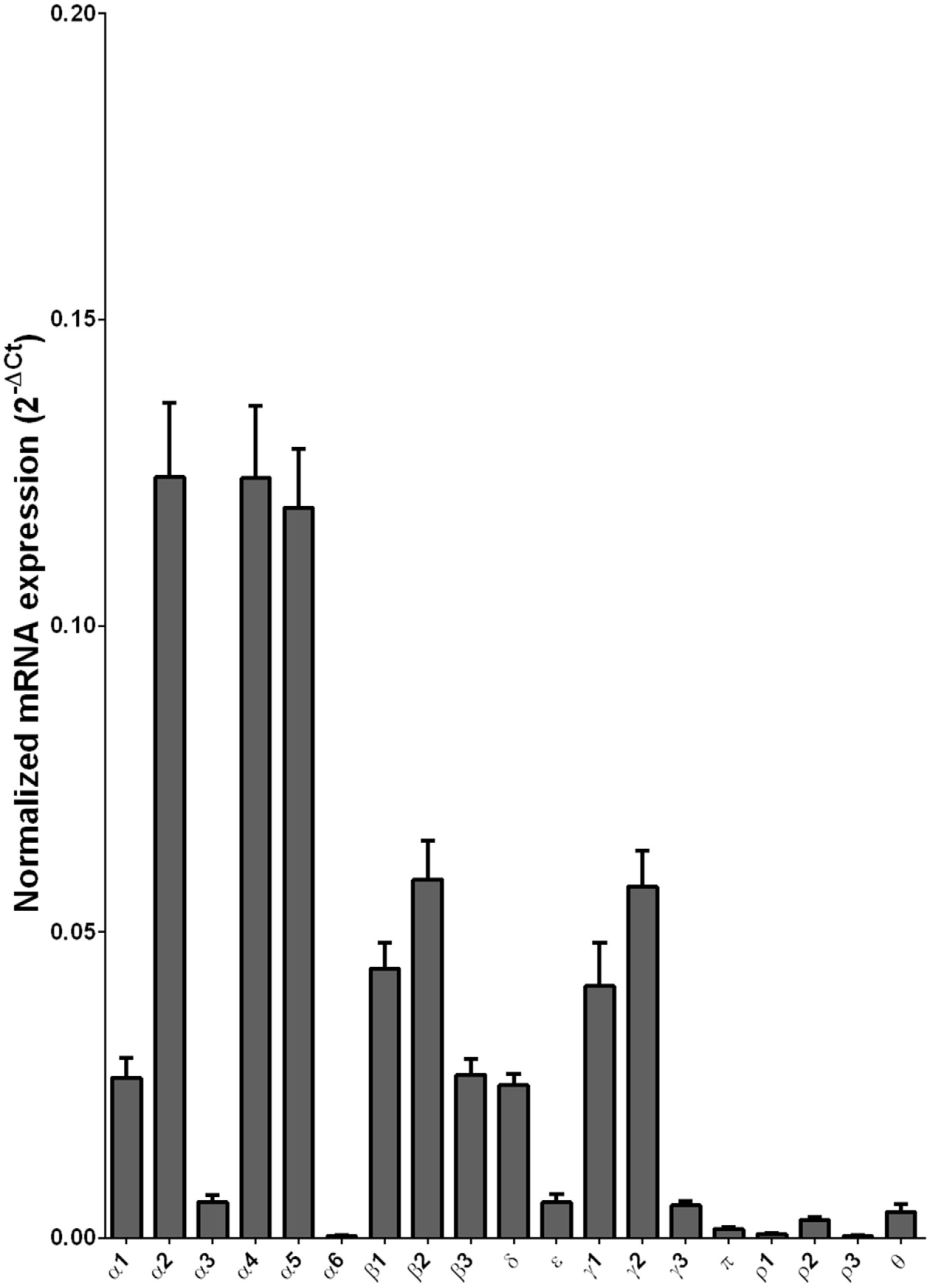
**Expression of GABA-A channel subunit mRNAs in the putamen of control subjects (*n* = 29)**. The mRNA level of each subunit was normalized to reference gene *ACTB* and presented as mean ± SEM.

**Figure 8:**
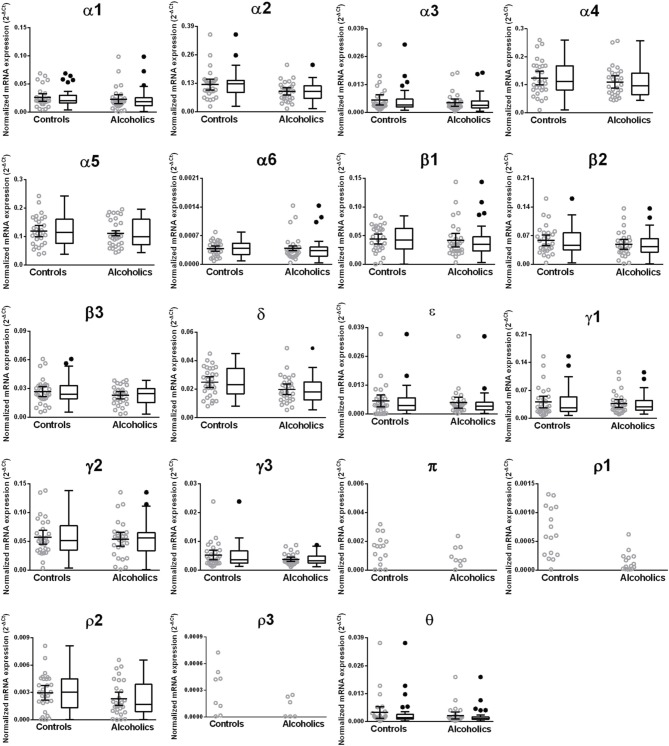
**Expression of GABA-A channel subunits mRNA in the putamen of controls (*n* = 29) and alcoholics (*n* = 29)**. Data from each group is presented as scatter dot plot (°) with mean and 95% confidence interval and box and whiskers plot with median and whiskers plotted by Tukey method to determine outliers (•, above or below the whiskers). Statistical analysis was performed by excluding outliers. One Way ANOVA with Bonferroni *post-hoc* test, α1, *df* = 47, *p* = 0.64; α2, *df* = 47, *p* = 0.12; α6, *df* = 47, *p* = 0.15; β 1, *df* = 47, *p* = 0.14; β 2, *df* = 47, *p* = 0.40; β 3, *df* = 47, *p* = 0.55; δ, *df* = 47, *p* = 0.093. Kruskal–Wallis ANOVA on ranks with Dunn's *post-hoc* test, α3, *H*_(1, 52)_ = 0.22, *p* = 0.64; α4, *H*_(1, 57)_ = 0.98, *p* = 0.32; α5, *H*_(1, 58)_ = 0.44, *p* = 0.51; ε, *H*_(1, 53)_ = 0.14, *p* = 0.71; γ1, *H*_(1, 53)_ = 0.27, *p* = 0.61; γ2, *H*_(1, 56)_ = 0.20, *p* = 0.65; γ3, *H*_(1, 55)_ = 1.51, *p* = 0.22; ρ 2, *H*_(1, 58)_ = 2.00, *p* = 0.16; θ, *H*_(1, 47)_ = 3.27, *p* = 0.071.

### Higher mRNA expression level of eleven glutamate and five GABA-A receptor subunits in the caudate nucleus as compared to the putamen in non-alcoholic controls

**Figure 9:**
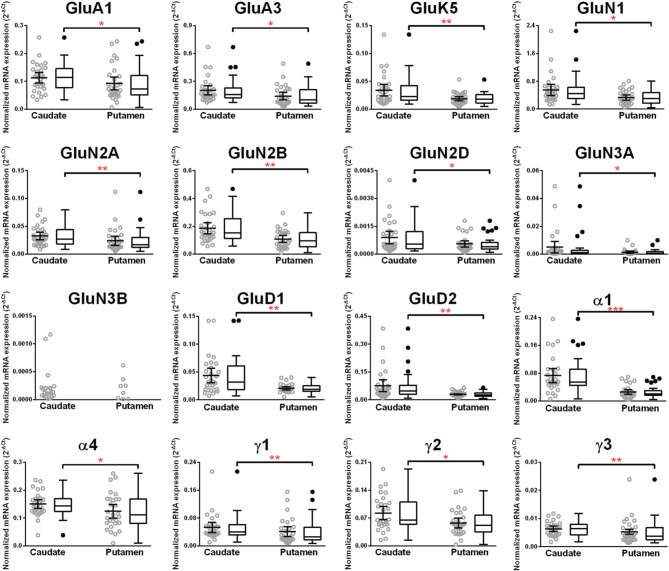
**Comparison of GABA-A and ionotropic glutamate receptor subunit mRNAs in the caudate and putamen of controls (*n* = 29)**. Data from each group is presented as scatter dot plot (°) with mean and 95% confidence interval and box and whiskers plot with median and whiskers plotted by Tukey method to determine outliers (•, above or below the whiskers). Statistical analysis was performed by excluding outliers. One Way ANOVA with Bonferroni *post-hoc* test, **GluA1**, *df* = 52, *p* = 0.04; α2, *df* = 52, *p* = 0.11; **α4**, *df* = 52, *p* = 0.019; α5, *df* = 52, *p* = 0.79; α6, *df* = 52, *p* = 0.45; β 1, *df* = 52, *p* = 0.086; β 2, *df* = 52, *p* = 0.48; β 3, *df* = 52, *p* = 0.96; δ, *df* = 52, *p* = 0.77. Kruskal–Wallis ANOVA on ranks with Dunn's *post-hoc* test, GluA2, *H*_(1, 55)_ = 0.48, *p* = 0.49; **GluA3**, *H*_(1, 54)_ = 4.99, *p* = 0.026; GluA4, *H*_(1, 56)_ = 3.34, *p* = 0.068; GluK1, *H*_(1, 56)_ = 2.6, *p* = 0.11; GluK2, *H*_(1, 54)_ = 2.48, *p* = 0.12; GluK3, *H*_(1, 53)_ = 1.42, *p* = 0.23; **GluK4**, *H*_(1, 53)_ = 0.0051, *p* = 0.94; **GluK5**, *H*_(1, 56)_ = 7.31, *p* = 0.0069; **GluN1**, *H*_(1, 56)_ = 5.23, *p* = 0.022; **GluN2A**, *H*_(1, 56)_ = 10.28, *p* = 0.0013; **GluN2B**, *H*_(1, 57)_ = 8.35, *p* = 0.0039; GluN2C, *H*_(1, 55)_ = 0.19, *p* = 0.66; **GluN2D**, *H*_(1, 52)_ = 5.06, *p* = 0.025; **GluN3A**, *H*_(1, 52)_ = 4.83, *p* = 0.028; **GluD1**, *H*_(1, 56)_ = 7.28, *p* = 0.007; **GluD2**, *H*_(1, 53)_ = 8.33, *p* = 0.0039; α1, *H*_(1, 49)_ = 23.62, *p* = 0.0000; α3, *H*_(1, 50)_ = 1.47, *p* = 0.23; ε, *H*_(1, 54)_ = 0.96, *p* = 0.33; γ 1, *H*_(1, 53)_ = 7.03 *p* = 0.008; γ2, *H*_(1, 58)_ = 6.31 *p* = 0.012; γ 3, *H*_(1, 57)_ = 7.45 *p* = 0.0063; ρ 2, *H*_(1, 57)_ = 2.20 *p* = 0.14; θ, *H*_(1, 50)_ = 0.85 *p* = 0.36. ^*^p < 0.05, ^**^p < 0.01, ^***^p < 0.001.

We compared the abundance of glutamate and GABA-A receptors in the caudate and the putamen by comparing the level of the specific mRNAs expressed by these structures (Figure 9, Table 1). Two AMPA (GluA1 and 3), one kainate (GluK5), five NMDA (GluN1, GluN2A, N2B, N2D, and N3A) and the two delta glutamate subunits were expressed at a higher level in the caudate (Figure 9) and the fold increase varied between 1.3 and 2.1 of the expression level in the putamen (Table 1). GluN3B expression level was similar in both regions but it was more frequently expressed in the caudate samples (76%) than in the putamen samples (28%). The GABA-A subunits that were expressed at higher levels in the caudate as compared to the putamen were the α1, α4, γ1, γ2, and γ3 with the fold change varying from 1.24 to 2.91. The largest difference between the two regions was observed for the α1 subunit that was expressed at about 3-fold higher level in the caudate. The GABA-A receptor α1, α2, and α3 subunits are often found in synapses, whereas the α4, α5, and α6 subunits are almost exclusively located outside of synapses where they contribute to tonic currents (Lindquist and Birnir, 2006; Jin et al., 2011b; Brickley and Mody, 2012). Interestingly, both in the caudate and putamen, the mRNA expression levels of the α4 and α5 subunits in this study were similar to or higher than those of the subunits normally found in synapses, suggesting a strong contribution of tonic inhibitory currents in the human striatal neurons.

## Discussion

The dorsal striatum has an important role in mediating goal-directed and habitual behavior and these functions are thought to reside within the caudate and putamen, respectively (Balleine et al., 2007). The caudate may, therefore, participate in e.g., goal-directed alcohol seeking whereas the putamen in the development of habitual alcohol use (Balleine and O'Doherty, 2010; Chen et al., 2011). Our results show that in the normal brain, 11 glutamate, and 5 GABA-A receptor genes were expressed at a significantly higher level in the caudate than in the putamen. Furthermore, in the brain tissue from alcoholics there were changes in the caudate but not the putamen in both the glutamate and GABA-A receptor mRNA levels. In the caudate, mRNA encoding one NMDA (GluN2A) and three GABA-A (δ, ε, ρ2) receptor subunits were down-regulated and one GABA-A subunit (γ1) was up-regulated. In agreement with other studies on human post-mortem samples (Lewohl et al., 1997, 2001; Dodd and Lewohl, 1998; Buckley et al., 2000, 2006; Mayfield et al., 2002; Buckley and Dodd, 2004; Dodd et al., 2004; Flatscher-Bader et al., 2005, 2006; Kalsi et al., 2009; Jin et al., 2011a, 2014a,b; Domart et al., 2012), our study further supports that distinct functional domains in the brain are differentially regulated and affected by ethanol.

Of the glutamate receptors, the NMDA receptors are most sensitive to ethanol and are partially inhibited by clinically relevant ethanol concentrations (starting from 20 mM) (Lovinger et al., 1989). How the ethanol's inhibition of the glutamate receptors translates to altered expression of the receptor subunits is not understood. As the NMDA receptors are involved in many physiological processes such as neuronal signal transduction, LTP, long-term depression (LTD), excitotoxicity (also in alcohol withdrawal phase), and neuronal survival, their altered function in alcoholics may directly or indirectly affect gene regulation in general and transcription of specific genes in some neurons. Epidemiological evidence indicates that more than 50% of the risk for becoming an alcoholic stems from genetic susceptibility (Kalsi et al., 2009). Many glutamate receptor genes have been identified as risk genes for alcoholism and alcohol-related phenotypes in human studies, including the GluN2A, the mRNA expression of which was now down-regulated in the caudate of alcoholics in this study (Wernicke et al., 2003; Petrakis et al., 2004; Rujescu et al., 2005; Kim et al., 2006; Preuss et al., 2006; Ray et al., 2009; Ridge et al., 2009; Domart et al., 2012; Jin et al., 2014a,b). NMDA receptors are tetrameric channels composed of two GluN1 subunits and either two GluN2 or a combination of GluN2 and GluN3 subunits (Smart and Paoletti, 2012). Importantly, the glutamate-binding GluN2 subunits mostly determine the synaptic function of NMDA receptors (Zhang and Luo, 2013), and if faithfully translated into protein, the low mRNA level in alcoholics would mean reduced NMDA receptor-mediated excitation in the caudate. The possible functional significance of the increased GluD1 and GluD2 subunit expression is not known.

The GABA-A receptors are chloride ion channels that are opened by GABA and when activated they decrease excitability of neurons. The GABA-A channels are pentameric and a change in the subunit composition can directly affect its cellular and sub-cellular location as well as physiological and pharmacological properties of the receptors (Olsen and Sieghart, 2009). Clinically relevant concentrations of alcohol may enhance GABA-A receptor-mediated currents in the brain (Olsen et al., 2007; Uusi-Oukari and Korpi, 2010). Many GABA-A receptor subunits have been correlated with alcoholism and alcohol-related phenotypes in human and animal studies, including the δ and γ1 subunits that we identified in this study (Devaud et al., 1995; Lewohl et al., 1997, 2001; Dodd and Lewohl, 1998; Matthews et al., 1998; Buckley et al., 2000, 2006; Buckley and Dodd, 2004; Liang et al., 2007; Jin et al., 2011a, 2014a). The most common GABA-A subunit in the mammalian brain is the α1 subunit, which can be located both at synapses and outside of synapses, whereas the α4 or α5 subunits are thought to have a more restrictive expression and be mostly located extrasynaptically (Jin et al., 2011b; Brickley and Mody, 2012). It was, therefore, surprising that high expression levels were observed for the α4 and α5 mRNAs in both the caudate and putamen. Our results suggest a strong tonic contribution of the GABA-A receptor function in these regions in addition to the synaptic GABAergic transmission. In agreement, also in mice large tonic currents have been detected in electrophysiological experiments on dorsal striatal neurons (Ade et al., 2008). The δ subunit is known to be exclusively located extrasynaptically, to combine in the forebrain with α4β subunits to form GABA-A receptors that have high affinity for GABA and that are modulated by ethanol (Brickley and Mody, 2012). In our study, the δ subunit was significantly down-regulated in the caudate from alcoholics indicating decreased tonic inhibition in the caudate region, which would be expected to increase the basic excitability of the neurons. In animal models, the δ subunit-containing receptors have been shown to regulate alcohol consumption and withdrawal (Mihalek et al., 2001) and their pharmacological activation has been linked to conditioned aversion (Vashchinkina et al., 2012), suggesting a correlation of the reduced δ subunit expression with low aversion and reduced withdrawal symptoms in alcoholics. The significance of the reductions of ε and ρ2 subunits in alcoholics is unknown, as their functions have been studied very little, with the ε subunit being the least conserved subunit between rodents and humans (Sinkkonen et al., 2000).

The GABA-A γ1 subunit was up-regulated in alcoholics in the caudate. Interestingly, GABA-A receptors that contain this subunit are potentiated by neurosteroids (Puia et al., 1993) and ethanol promotes release of endogenous neurosteroids (Biggio et al., 2007) thus enhancing GABA-A currents in neurons. Chronic ethanol administration in rodents significantly increases expression of the γ1 subunit in the cerebral cortex (Devaud et al., 1995) and hippocampus (Cagetti et al., 2003) and a similar increase was recorded in hippocampal post-mortem brain samples from alcoholics (Jin et al., 2011a). Factorial analysis of global GABAergic gene expression in human brains has further identified the α2β1γ1 gene cluster on chromosome 4 as being influenced by chronic alcohol exposure (Enoch et al., 2013). Therefore, increased sensitivity to neurosteroids of GABA-A receptors may be associated with alcoholism.

Importantly, although alcohol may affect levels of glutamate and GABA-A receptors in the brain by a multitude of mechanisms, our results in this study and recent studies involving the hippocampus, prefrontal cortex, orbitofrontal cortex and amygdala (Jin et al., 2011a, 2014a,b) suggest a strict regional specificity of the effects of alcohol on the mRNA expression of ionotropic glutamate and GABA-A receptor subunits.

Chronic, heavy consumption of alcohol by humans has been shown to lead to impairment of executive and cognitive functions that require normal prefrontal cortical function (Goldstein et al., 2004; Crego et al., 2010). We have previously shown in post-mortem samples from alcoholics that the mRNA expression levels of glutamate and GABA-A receptors in the dorsal-lateral prefrontal cortex (DL-PFC) are not altered from that of normal controls (Jin et al., 2014b). This is in contrast to the present study where mRNA expression of specific glutamate and GABA receptor subunits were altered in the caudate of the alcoholics. Imaging studies have demonstrated connectivity and significant co-activation between caudate and higher level cognitive areas like the DL-PFC, rostral anterior cingulate and inferior frontal gyri (Strafella et al., 2003; Postuma and Dagher, 2006; Grahn et al., 2008). In contrast, the putamen links more to the primary cortical motor areas (Postuma and Dagher, 2006; Grahn et al., 2008). Recent neurobiological ideas of alcoholism (Depoy et al., 2013) suggest that there may be diminished executive effects on alcohol seeking and behavior and, rather, a shift to greater striatal control over goal-directed rewarded behaviors that may be critical in progress of becoming an alcoholic. Our finding of alterations in excitatory and inhibitory receptor subunit mRNA expression in the caudate might constitute a part of the underlying neuropathological mechanisms of the shift in striatal function of alcoholics.

## Author contributions

Igor Bazov, Olga Kononenko, Georgy Bakalkin obtained the material and made the RNA from the tissue. Amol K. Bhandage, Zhe Jin, Esa R. Korpi, and Bryndis Birnir designed the experiments. Amol K. Bhandage and Zhe Jin designed primers and ran the qPCR. Amol K. Bhandage, and Zhe Jin made the figures and did the statistical analysis. Bryndis Birnir wrote the paper that was edited by Amol K. Bhandage, Zhe Jin, and Esa R. Korpi and then commented on by other authors.

### Conflict of interest statement

The work was mainly supported by Swedish Research Council grant to Bryndis Birnir and, in part, from the Swedish Science Research Councils FAS, VR, and FORMAS to Georgy Bakalkin. The funders had no role in study design, data collection and analysis, decision to publish or preparation of the manuscript. The authors declare that the research was conducted in the absence of any commercial or financial relationships that could be construed as a potential conflict of interest.

## References

[B1] Ade K. K. Janssen M. J. Ortinski P. I. Vicini S. (2008). Differential tonic GABA conductances in striatal medium spiny neurons. J. Neurosci. 28, 1185–1197. 10.1523/JNEUROSCI.3908-07.2008 18234896 PMC6671393

[B2] Alexander G. E. Delong M. R. Strick P. L. (1986). Parallel organization of functionally segregated circuits linking basal ganglia and cortex. Annu. Rev. Neurosci. 9, 357–381. 10.1146/annurev.ne.09.030186.002041 3085570

[B3] Balleine B. W. Delgado M. R. Hikosaka O. (2007). The role of the dorsal striatum in reward and decision-making. J. Neurosci. 27, 8161–8165. 10.1523/JNEUROSCI.1554-07.2007 17670959 PMC6673072

[B4] Balleine B. W. O'Doherty J. P. (2010). Human and rodent homologies in action control: corticostriatal determinants of goal-directed and habitual action. Neuropsychopharmacology 35, 48–69. 10.1038/npp.2009.131 19776734 PMC3055420

[B5] Baur R. Kaur K. H. Sigel E. (2009). Structure of alpha6 beta3 delta GABA(A) receptors and their lack of ethanol sensitivity. J. Neurochem. 111, 1172–1181. 10.1111/j.1471-4159.2009.06387.x 19765192

[B81] Bazov I. Kononenko O. Watanabe H. Kuntic V. Sarkisyan D. Taqi M. M.. (2011). The endogenous opioid system in human alcoholics: molecular adaptations in brain areas involved in cognitive control of addiction. Addict. Biol. 18, 161–169. 10.1111/j.1369-1600.2011.00366.x 21955155

[B6] Biggio G. Concas A. Follesa P. Sanna E. Serra M. (2007). Stress, ethanol, and neuroactive steroids. Pharmacol. Ther. 116, 140–171. 10.1016/j.pharmthera.2007.04.005 17555824 PMC3000046

[B7] Blitzer R. D. Gil O. Landau E. M. (1990). Long-term potentiation in rat hippocampus is inhibited by low concentrations of ethanol. Brain Res. 537, 203–208. 10.1016/0006-8993(90)90359-J 2150775

[B8] Borghese C. M. Storustovu S. Ebert B. Herd M. B. Belelli D. Lambert J. J.. (2006). The delta subunit of gamma-aminobutyric acid type A receptors does not confer sensitivity to low concentrations of ethanol. J. Pharmacol. Exp. Ther. 316, 1360–1368. 10.1124/jpet.105.092452 16272217

[B9] Brickley S. G. Mody I. (2012). Extrasynaptic GABA(A) receptors: their function in the CNS and implications for disease. Neuron 73, 23–34. 10.1016/j.neuron.2011.12.012 22243744 PMC3399243

[B10] Buckley S. T. Dodd P. R. (2004). GABAA receptor beta subunit mRNA expression in the human alcoholic brain. Neurochem. Int. 45, 1011–1020. 10.1016/j.neuint.2004.05.009 15337300

[B11] Buckley S. T. Eckert A. L. Dodd P. R. (2000). Expression and distribution of GABAA receptor subtypes in human alcoholic cerebral cortex. Ann. N.Y. Acad. Sci. 914, 58–64. 10.1111/j.1749-6632.2000.tb05183.x 11085308

[B12] Buckley S. T. Foley P. F. Innes D. J. Loh El W. Shen Y. Williams S. M.. (2006). GABA(A) receptor beta isoform protein expression in human alcoholic brain: interaction with genotype. Neurochem. Int. 49, 557–567. 10.1016/j.neuint.2006.04.008 16766085

[B13] Cagetti E. Liang J. Spigelman I. Olsen R. W. (2003). Withdrawal from chronic intermittent ethanol treatment changes subunit composition, reduces synaptic function, and decreases behavioral responses to positive allosteric modulators of GABAA receptors. Mol. Pharmacol. 63, 53–64. 10.1124/mol.63.1.53 12488536

[B14] Chen G. Cuzon Carlson V. C. Wang J. Beck A. Heinz A. Ron D.. (2011). Striatal involvement in human alcoholism and alcohol consumption, and withdrawal in animal models. Alcohol. Clin. Exp. Res. 35, 1739–1748. 10.1111/j.1530-0277.2011.01520.x 21615425 PMC3276303

[B15] Crabbe J. C. Kendler K. S. Hitzemann R. J. (2013). Modeling the diagnostic criteria for alcohol dependence with genetic animal models. Curr. Top. Behav. Neurosci. 13, 187–221. 10.1007/978-3-642-28720-6_162 21910077 PMC3371181

[B16] Crego A. Rodriguez-Holguin S. Parada M. Mota N. Corral M. Cadaveira F. (2010). Reduced anterior prefrontal cortex activation in young binge drinkers during a visual working memory task. Drug Alcohol Depend. 109, 45–56. 10.1016/j.drugalcdep.2009.11.020 20079980

[B76] Denisov V. Strong V. Walter V. Gingrich J. Wintz H. (2008). Development and validation of RQI: an RNA quality indicator for the experion automated electrophoresis system, in Bio-Rad Technology Note (Hercules, CA).

[B17] Depoy L. Daut R. Brigman J. L. Macpherson K. Crowley N. Gunduz-Cinar O.. (2013). Chronic alcohol produces neuroadaptations to prime dorsal striatal learning. Proc. Natl. Acad. Sci. U.S.A. 110, 14783–14788. 10.1073/pnas.1308198110 23959891 PMC3767559

[B18] Devaud L. L. Smith F. D. Grayson D. R. Morrow A. L. (1995). Chronic ethanol consumption differentially alters the expression of gamma-aminobutyric acidA receptor subunit mRNAs in rat cerebral cortex: competitive, quantitative reverse transcriptase-polymerase chain reaction analysis. Mol. Pharmacol. 48, 861–868. 7476917

[B19] Dodd P. R. Foley P. F. Buckley S. T. Eckert A. L. Innes D. J. (2004). Genes and gene expression in the brain of the alcoholic. Addict. Behav. 29, 1295–1309. 10.1016/j.addbeh.2004.06.010 15345266

[B20] Dodd P. R. Lewohl J. M. (1998). Cell death mediated by amino acid transmitter receptors in human alcoholic brain damage: conflicts in the evidence. Ann. N.Y. Acad. Sci. 844, 50–58. 10.1111/j.1749-6632.1998.tb08221.x 9668664

[B21] Domart M. C. Benyamina A. Lemoine A. Bourgain C. Blecha L. Debuire B.. (2012). Association between a polymorphism in the promoter of a glutamate receptor subunit gene (GRIN2A) and alcoholism. Addict. Biol. 17, 783–785. 10.1111/j.1369-1600.2011.00321.x 21507155

[B22] Enoch M. A. Baghal B. Yuan Q. Goldman D. (2013). A factor analysis of global GABAergic gene expression in human brain identifies specificity in response to chronic alcohol and cocaine exposure. PLoS ONE 8:e64014. 10.1371/journal.pone.0064014 23717525 PMC3661725

[B23] Flatscher-Bader T. Van Der Brug M. Hwang J. W. Gochee P. A. Matsumoto I. Niwa S.. (2005). Alcohol-responsive genes in the frontal cortex and nucleus accumbens of human alcoholics. J. Neurochem. 93, 359–370. 10.1111/j.1471-4159.2004.03021.x 15816859

[B24] Flatscher-Bader T. Van Der Brug M. P. Landis N. Hwang J. W. Harrison E. Wilce P. A. (2006). Comparative gene expression in brain regions of human alcoholics. Genes Brain Behav. 5(Suppl 1), 78–84. 10.1111/j.1601-183X.2006.00197.x 16417620

[B77] Fleige S. Pfaffl M. W. (2006). RNA integrity and the effect on the real-time qRT-PCR performance. Mol. Aspects Med. 27, 126–139. 10.1016/j.mam.2005.12.003 16469371

[B82] Fleige S. Walf V. Huch S. Prgomet C. Sehm J. Pfaffl M. W. (2006). Comparison of relative mRNA quantification models and the impact of RNA integrity in quantitative real-time RT-PCR. Biotechnol. Lett. 28, 1601–1613. 10.1007/s10529-006-9127-2 16900335

[B25] Gass J. T. Olive M. F. (2008). Glutamatergic substrates of drug addiction and alcoholism. Biochem. Pharmacol. 75, 218–265. 10.1016/j.bcp.2007.06.039 17706608 PMC2239014

[B26] Givens B. McMahon K. (1995). Ethanol suppresses the induction of long-term potentiation in vivo. Brain Res. 688, 27–33. 10.1016/0006-8993(95)00499-G 8542319

[B27] Goldstein R. Z. Leskovjan A. C. Hoff A. L. Hitzemann R. Bashan F. Khalsa S. S.. (2004). Severity of neuropsychological impairment in cocaine and alcohol addiction: association with metabolism in the prefrontal cortex. Neuropsychologia 42, 1447–1458. 10.1016/j.neuropsychologia.2004.04.002 15246283

[B28] Grahn J. A. Parkinson J. A. Owen A. M. (2008). The cognitive functions of the caudate nucleus. Prog. Neurobiol. 86, 141–155. 10.1016/j.pneurobio.2008.09.004 18824075

[B83] Gubelmann C. Gattiker A. Massouras A. Hens K. David F. Decouttere F.. (2011). GETPrime: a gene- or transcript-specific primer database for quantitative real-time PCR. Database (Oxford), 2011:bar040. 10.1093/database/bar040 21917859 PMC3173022

[B29] Hardwick R. M. Rottschy C. Miall R. C. Eickhoff S. B. (2013). A quantitative meta-analysis and review of motor learning in the human brain. Neuroimage 67, 283–297. 10.1016/j.neuroimage.2012.11.020 23194819 PMC3555187

[B30] Harper C. (1998). The neuropathology of alcohol-specific brain damage, or does alcohol damage the brain? J. Neuropathol. Exp. Neurol. 57, 101–110. 10.1097/00005072-199802000-00001 9600202

[B31] Harris R. A. Trudell J. R. Mihic S. J. (2008). Ethanol's molecular targets. Sci Signal 1:re7. 10.1126/scisignal.128re7 18632551 PMC2671803

[B32] Jernigan T. L. Butters N. Ditraglia G. Schafer K. Smith T. Irwin M.. (1991). Reduced cerebral grey matter observed in alcoholics using magnetic resonance imaging. Alcohol. Clin. Exp. Res. 15, 418–427. 10.1111/j.1530-0277.1991.tb00540.x 1877728

[B33] Jin Z. Bazov I. Kononenko O. Korpi E. R. Bakalkin G. Birnir B. (2011a). Selective changes of GABA(A) channel subunit mRNAs in the hippocampus and orbitofrontal cortex but not in prefrontal cortex of human alcoholics. Front. Cell. Neurosci. 5:30. 10.3389/fncel.2011.00030 22319468 PMC3249692

[B34] Jin Z. Bhandage A. K. Bazov I. Kononenko O. Bakalkin G. Korpi E. R.. (2014a). Expression of specific ionotropic glutamate and GABA-A receptor subunits is decreased in central amygdala of alcoholics. Front. Cell. Neurosci. 8:288. 10.3389/fncel.2014.00288 25278838 PMC4165314

[B35] Jin Z. Bhandage A. K. Bazov I. Kononenko O. Bakalkin G. Korpi E. R.. (2014b). Selective increases of AMPA, NMDA, and kainate receptor subunit mRNAs in the hippocampus and orbitofrontal cortex but not in prefrontal cortex of human alcoholics. Front. Cell. Neurosci. 8:11. 10.3389/fncel.2014.00011 24523671 PMC3905203

[B36] Jin Z. Jin Y. Kumar-Mendu S. Degerman E. Groop L. Birnir B. (2011b). Insulin reduces neuronal excitability by turning on GABA(A) channels that generate tonic current. PLoS ONE 6:e16188. 10.1371/journal.pone.0016188 21264261 PMC3021545

[B79] Johansson S. Fuchs A. Okvist A. Karimi M. Harper C. Garrick T.. (2007). Validation of endogenous controls for quantitative gene expression analysis: application on brain cortices of human chronic alcoholics. Brain Res. 1132, 20–28. 10.1016/j.brainres.2006.11.026 17188656

[B37] Kalsi G. Prescott C. A. Kendler K. S. Riley B. P. (2009). Unraveling the molecular mechanisms of alcohol dependence. Trends Genet. 25, 49–55. 10.1016/j.tig.2008.10.005 19010566

[B38] Kim J. H. Park M. Yang S. Y. Jeong B. S. Yoo H. J. Kim J. W.. (2006). Association study of polymorphisms in N-methyl-D-aspartate receptor 2B subunits (GRIN2B) gene with Korean alcoholism. Neurosci. Res. 56, 220–223. 10.1016/j.neures.2006.06.013 16911840

[B39] Korpi E. R. Debus F. Linden A. M. Malecot C. Leppa E. Vekovischeva O.. (2007). Does ethanol act preferentially via selected brain GABAA receptor subtypes? the current evidence is ambiguous. Alcohol 41, 163–176. 10.1016/j.alcohol.2007.03.007 17591542

[B80] Kuzmin A. Bazov I. Sheedy D. Garrick T. Harper C. Bakalkin G. (2009). Expression of pronociceptin and its receptor is downregulated in the brain of human alcoholics. Brain Res. 1305(Suppl.), S80–S85. 10.1016/j.brainres.2009.05.067 19501074 PMC3391552

[B40] Lewohl J. M. Crane D. I. Dodd P. R. (1997). Expression of the alpha 1, alpha 2 and alpha 3 isoforms of the GABAA receptor in human alcoholic brain. Brain Res. 751, 102–112. 10.1016/S0006-8993(96)01396-0 9098573

[B41] Lewohl J. M. Huygens F. Crane D. I. Dodd P. R. (2001). GABA(A) receptor alpha-subunit proteins in human chronic alcoholics. J. Neurochem. 78, 424–434. 10.1046/j.1471-4159.2001.00414.x 11483645

[B42] Liang J. Suryanarayanan A. Abriam A. Snyder B. Olsen R. W. Spigelman I. (2007). Mechanisms of reversible GABAA receptor plasticity after ethanol intoxication. J. Neurosci. 27, 12367–12377. 10.1523/JNEUROSCI.2786-07.2007 17989301 PMC6673253

[B43] Lindquist C. E. Birnir B. (2006). Graded response to GABA by native extrasynaptic GABA receptors. J. Neurochem. 97, 1349–1356. 10.1111/j.1471-4159.2006.03811.x 16573642

[B44] Lovinger D. M. Roberto M. (2013). Synaptic effects induced by alcohol. Curr. Top. Behav. Neurosci. 13, 31–86. 10.1007/978-3-642-28720-6_143 21786203 PMC4791588

[B45] Lovinger D. M. White G. Weight F. F. (1989). Ethanol inhibits NMDA-activated ion current in hippocampal neurons. Science 243, 1721–1724. 10.1126/science.2467382 2467382

[B46] Matthews D. B. Devaud L. L. Fritschy J. M. Sieghart W. Morrow A. L. (1998). Differential regulation of GABA(A) receptor gene expression by ethanol in the rat hippocampus versus cerebral cortex. J. Neurochem. 70, 1160–1166. 10.1046/j.1471-4159.1998.70031160.x 9489737

[B47] Mayfield R. D. Lewohl J. M. Dodd P. R. Herlihy A. Liu J. Harris R. A. (2002). Patterns of gene expression are altered in the frontal and motor cortices of human alcoholics. J. Neurochem. 81, 802–813. 10.1046/j.1471-4159.2002.00860.x 12065639

[B48] Mihalek R. M. Bowers B. J. Wehner J. M. Kralic J. E. Vandoren M. J. Morrow A. L.. (2001). GABA(A)-receptor delta subunit knockout mice have multiple defects in behavioral responses to ethanol. Alcohol. Clin. Exp. Res. 25, 1708–1718. 10.1111/j.1530-0277.2001.tb02179.x 11781502

[B49] Mihic S. J. Ye Q. Wick M. J. Koltchine V. V. Krasowski M. D. Finn S. E.. (1997). Sites of alcohol and volatile anaesthetic action on GABA(A) and glycine receptors. Nature 389, 385–389. 10.1038/38738 9311780

[B50] Morrisett R. A. Swartzwelder H. S. (1993). Attenuation of hippocampal long-term potentiation by ethanol: a patch-clamp analysis of glutamatergic and GABAergic mechanisms. J. Neurosci. 13, 2264–2272. 8478698 10.1523/JNEUROSCI.13-05-02264.1993 PMC6576561

[B51] Moykkynen T. Korpi E. R. (2012). Acute effects of ethanol on glutamate receptors. Basic Clin. Pharmacol. Toxicol. 111, 4–13. 10.1111/j.1742-7843.2012.00879.x 22429661

[B52] Olsen R. W. Hanchar H. J. Meera P. Wallner M. (2007). GABAA receptor subtypes: the “one glass of wine” receptors. Alcohol 41, 201–209. 10.1016/j.alcohol.2007.04.006 17591543 PMC2852584

[B53] Olsen R. W. Sieghart W. (2009). GABA A receptors: subtypes provide diversity of function and pharmacology. Neuropharmacology 56, 141–148. 10.1016/j.neuropharm.2008.07.045 18760291 PMC3525320

[B54] Petrakis I. L. Limoncelli D. Gueorguieva R. Jatlow P. Boutros N. N. Trevisan L.. (2004). Altered NMDA glutamate receptor antagonist response in individuals with a family vulnerability to alcoholism. Am. J. Psychiatry 161, 1776–1782. 10.1176/appi.ajp.161.10.1776 15465973

[B55] Postuma R. B. Dagher A. (2006). Basal ganglia functional connectivity based on a meta-analysis of 126 positron emission tomography and functional magnetic resonance imaging publications. Cereb. Cortex 16, 1508–1521. 10.1093/cercor/bhj088 16373457

[B56] Preuss U. W. Zill P. Koller G. Bondy B. Hesselbrock V. Soyka M. (2006). Ionotropic glutamate receptor gene GRIK3 SER310ALA functional polymorphism is related to delirium tremens in alcoholics. Pharmacogenomics J. 6, 34–41. 10.1038/sj.tpj.6500343 16314883

[B57] Puia G. Ducic I. Vicini S. Costa E. (1993). Does neurosteroid modulatory efficacy depend on GABAA receptor subunit composition? Recept. Channels 1, 135–142. 8081717

[B58] Ray L. A. Miranda R. Jr. Mackillop J. McGeary J. Tidey J. W. Rohsenow D. J.. (2009). A preliminary pharmacogenetic investigation of adverse events from topiramate in heavy drinkers. Exp. Clin. Psychopharmacol. 17, 122–129. 10.1037/a0015700 19331489 PMC3682424

[B59] Ridge J. P. Ho A. M. Dodd P. R. (2009). Sex differences in NMDA receptor expression in human alcoholics. Alcohol Alcohol. 44, 594–601. 10.1093/alcalc/agp052 19736238 PMC2781764

[B60] Rujescu D. Soyka M. Dahmen N. Preuss U. Hartmann A. M. Giegling I.. (2005). GRIN1 locus may modify the susceptibility to seizures during alcohol withdrawal. Am. J. Med. Genet. B Neuropsychiatr. Genet. 133B, 85–87. 10.1002/ajmg.b.30112 15635650

[B78] Schmittgen T. D. Livak K. J. (2008). Analyzing real-time PCR data by the comparative C(T) method. Nat. Protoc. 3, 1101–1108. 10.1038/nprot.2008.73 18546601

[B61] Semyanov A. Walker M. C. Kullmann D. M. Silver R. A. (2004). Tonically active GABA A receptors: modulating gain and maintaining the tone. Trends Neurosci. 27, 262–269. 10.1016/j.tins.2004.03.005 15111008

[B62] Sheedy D. Garrick T. Dedova I. Hunt C. Miller R. Sundqvist N.. (2008). An Australian brain bank: a critical investment with a high return! Cell Tissue Bank. 9, 205–216. 10.1007/s10561-008-9076-1 18543078 PMC3391553

[B63] Siggins G. R. Roberto M. Nie Z. (2005). The tipsy terminal: presynaptic effects of ethanol. Pharmacol. Ther. 107, 80–98. 10.1016/j.pharmthera.2005.01.006 15963352

[B64] Sinkkonen S. T. Hanna M. C. Kirkness E. F. Korpi E. R. (2000). GABA(A) receptor epsilon and theta subunits display unusual structural variation between species and are enriched in the rat locus ceruleus. J. Neurosci. 20, 3588–3595. 10804200 10.1523/JNEUROSCI.20-10-03588.2000 PMC6772669

[B65] Smart T. G. Paoletti P. (2012). Synaptic neurotransmitter-gated receptors. Cold Spring Harb. Perspect. Biol. 4:a009662. 10.1101/cshperspect.a009662 22233560 PMC3282413

[B66] Spanagel R. (2009). Alcoholism: a systems approach from molecular physiology to addictive behavior. Physiol. Rev. 89, 649–705. 10.1152/physrev.00013.2008 19342616

[B67] Strafella A. P. Paus T. Fraraccio M. Dagher A. (2003). Striatal dopamine release induced by repetitive transcranial magnetic stimulation of the human motor cortex. Brain 126, 2609–2615. 10.1093/brain/awg268 12937078

[B68] Sundstrom-Poromaa I. Smith D. H. Gong Q. H. Sabado T. N. Li X. Light A.. (2002). Hormonally regulated alpha(4)beta(2)delta GABA(A) receptors are a target for alcohol. Nat. Neurosci. 5, 721–722. 10.1038/nn888 12118257 PMC2887346

[B69] Traynelis S. F. Wollmuth L. P. McBain C. J. Menniti F. S. Vance K. M. Ogden K. K.. (2010). Glutamate receptor ion channels: structure, regulation, and function. Pharmacol. Rev. 62, 405–496. 10.1124/pr.109.002451 20716669 PMC2964903

[B70] Uusi-Oukari M. Korpi E. R. (2010). Regulation of GABA(A) receptor subunit expression by pharmacological agents. Pharmacol. Rev. 62, 97–135. 10.1124/pr.109.002063 20123953

[B71] Vashchinkina E. Panhelainen A. Vekovischeva O. Y. Aitta-Aho T. Ebert B. Ator N. A. (2012). GABA site agonist gaboxadol induces addiction-predicting persistent changes in ventral tegmental area dopamine neurons but is not rewarding in mice or baboons. J. Neurosci. 32, 5310–5320 10.1523/JNEUROSCI.4697-11.2012 22496576 PMC6622081

[B72] Wallner M. Hanchar H. J. Olsen R. W. (2003). Ethanol enhances alpha 4 beta 3 delta and alpha 6 beta 3 delta gamma-aminobutyric acid type A receptors at low concentrations known to affect humans. Proc. Natl. Acad. Sci. U.S.A. 100, 15218–15223. 10.1073/pnas.2435171100 14625373 PMC299963

[B73] Wei W. Faria L. C. Mody I. (2004). Low ethanol concentrations selectively augment the tonic inhibition mediated by delta subunit-containing GABAA receptors in hippocampal neurons. J. Neurosci. 24, 8379–8382. 10.1523/JNEUROSCI.2040-04.2004 15385620 PMC6729680

[B74] Wernicke C. Samochowiec J. Schmidt L. G. Winterer G. Smolka M. Kucharska-Mazur J.. (2003). Polymorphisms in the N-methyl-D-aspartate receptor 1 and 2B subunits are associated with alcoholism-related traits. Biol. Psychiatry 54, 922–928. 10.1016/S0006-3223(03)00072-6 14573320

[B75] Zhang X. M. Luo J. H. (2013). GluN2A versus GluN2B: twins, but quite different. Neurosci. Bull. 29, 761–772. 10.1007/s12264-013-1336-9 23604599 PMC5561830

