# Supplementary material for: GABA-A and NMDA receptor subunit mRNA expression is altered in the caudate but not the putamen of the postmortem brains of alcoholics
Source: Front Cell Neurosci. 2014 Dec 5;8:415. doi: 10.3389/fncel.2014.00415 (PMC4257153; doi:10.3389/fncel.2014.00415)
Supplement: Supplementary file 1 [file Table1.DOCX]

**Table S1.** Sample demographic information.

| **Characteristics** | **Controls** | **Alcoholics** | ***p* Value** |
| --- | --- | --- | --- |
| Number | 29 | 29 |  |
| Age (years) | 57±2 | 58±2 | 0.91 |
| PMI (h) | 30±2.8 | 36±3.1 | 0.61 |
| Brain pH | 6.6±0.05 | 6.5±0.05 | 0.26 |
| RNA quality indicator | 7.5±0.20 | 7.3±0.21 | 0.57 |
| Smoking history* | 17(71%) S, 7(29%) NS  Available:24 | 20(77%) S, 6(23%) NS  Available:26 | 0.13 |

PMI, post-mortem interval; S, smoker; NS, non-smoker. Age, PMI, brain pH, and RNA quality indicator are shown as mean±SE, and the difference between controls and alcoholics was tested with Student’s t-test or Mann–Whitney U-test.

*Smoking histories are not available for all subjects. The proportion of smokers and non-smokers between controls and alcoholics was tested with Fisher’s exact test.

**Table S2.** Demographic data of controls and alcoholics

| **Subject**  **No.** | **Age**  **(Years)** | **PMI**  **(Hours)** | **Brain pH** | **Smoking**  **History** | **RQI** | **Ethnicity** | **Liver path** | **Cause of death** | **History of psychiatric illness** |
| --- | --- | --- | --- | --- | --- | --- | --- | --- | --- |
| **CONTROLS** | | | | | | | | | |
| **1** | 36 | 34 | 6.67 | Yes | 5.9 | Caucasian |  | Cardiac | No |
| **2** | 37 | 14.5 | 6.46 | No | 7.7 | Caucasian | Normal | Cardiac | No |
| **3** | 63 | 72 | 6.9 | Ex-smoker | 8.1 | Caucasian | Congestion | Cardiac: Severe coronary artery atherosclerosis | No |
| **4** | 82 | 23.5 | 6.4 | NA | 5.9 | Caucasian | Congestion | Infection: Multiple Organ Failure - Sepsis due to Small Bowel Perforation | No |
| **5** | 43 | 66 | 6.2 | NA | 6.0 | Caucasian | Congestion | Respiratory: Aspiration pneumonia. Complicating multi drug toxicity | No |
| **6** | 69 | 16 | 6.6 | Yes | 8.2 | Caucasian | Normal | Cardiac: Atherosclerotic cardiovascular disease | No |
| **7** | 46 | 25 | 6.65 | NA | 8.5 | Caucasian | Steatosis | Cardiac: Mitral valve prolapse | No |
| **8** | 57 | 18 | 6.6 | Ex-smoker | 7.8 | Caucasian | Congestion | Cardiac: Ischaemic heart disease | No |
| **9** | 55 | 20 | 6.5 | Ex-smoker | 8.6 | Middle Eastern | Steatosis | Cardiac | No |
| **10** | 56 | 37 | 6.76 | Yes | 8.2 | Caucasian | inflamation | Cardiac: Left ventricular (LV) scarring, hypertension and cardiomegaly | No |
| **11** | 47 | 38 | 6.74 | Yes | 8.6 | Caucasian | Steatosis | Cardiac | No |
| **12** | 50 | 30 | 6.37 | Yes | 7.2 | Caucasian | Normal | Cardiac: Ischaemic heart disease | No |
| **13** | 53 | 16 | 6.5 | Yes | 8.9 | Caucasian | Congestion | Cardiac | No |
| **14** | 53 | 27 | 6.64 | NA | 7.7 | Caucasian | Congestion | Cardiac | No |
| **15** | 54 | 28 | 6.38 | Yes | 8.3 | Caucasian | Congestion | Cardiac | No |
| **16** | 50 | 19 | 6.26 | Ex-smoker | 7.8 | Caucasian | Congestion | Cardiac | No |
| **17** | 58 | 28 | 5.92 | Yes | 5.7 | Caucasian |  | Cardiac | No |
| **18** | 59 | 40 | 6.53 | Ex-smoker | 6.4 | Caucasian | Normal | Cardiac | No |
| **19** | 59 | 43 | 6.69 | Yes | 8.4 | Caucasian | Steatosis | Cardiac | No |
| **20** | 60 | 21.5 | 6.66 | No | 7.8 | Caucasian | Steatosis | Cardiac | No |
| **21** | 60 | 13 | 6.59 | NA | 8.9 | Caucasian | Congestion | Cardiac | No |
| **22** | 60 | 25 | 6.7 | No | 6.0 | Caucasian |  | Infection | No |
| **23** | 61 | 27.5 | 6.25 | No | 5.5 | Caucasian | Congestion | Cardiac | No |
| **24** | 63 | 24 | 6.94 | Yes | 7.3 | Caucasian | Steatosis | Cardiac | No |
| **25** | 64 | 9.5 | 6.94 | Yes | 8.3 | Caucasian | Steatosis | Cardiac | No |
| **26** | 68 | 45.5 | 6.12 | No | 6.0 | Caucasian | Congestion | Cardiac | No |
| **27** | 69 | 52 | 6.95 | No | 8.6 | Caucasian | Normal | Cardiac | No |
| **28** | 73 | 38.5 | 6.28 | Ex-smoker | 8.1 | Caucasian | Steatosis | Cardiac | No |
| **29** | 54 | 29 | 6.8 | No | 7.3 | Asian | Steatosis | Cardiac | No |
| **ALCOHOLICS** | | | | | | | | | |
| **1** | 37 | 17 | 6.33 | No | 6.6 | Caucasian | Cirrhosis | Toxicity | No |
| **2** | 41 | 54 | 6.7 | Yes | 8.2 | Caucasian | Congestion | Neurological: Epilepsy | No |
| **3** | 65 | 72 | 6.88 | Yes | 7.1 | Caucasian | Congestion | Stroke | History of depression over 10 year-(does not meet criteria for DSMIV - Major Depressive Disorder) |
| **4** | 73 | 43.5 | 6.59 | No | 7.0 | Caucasian | Congestion | Cardiac | Long history of obsessive compulsive as well as depressive behaviour underlying a 'drinking problem'. |
| **5** | 42 | 41 | 6.5 | No | 7.7 | Caucasian | Steatosis | Toxicity: Combined Bromoxynil intox and alcohol toxicity | No |
| **6** | 57 | 43 | 6.46 | Yes | 8.6 | Caucasian | Cirrhosis | Respiratory | No |
| **7** | 59 | 24 | 6.57 | No | 6.6 | Caucasian | Steatosis | Cardiac: Cardiomyopathy | No |
| **8** | 56 | 22 | 6.52 | Yes | 8.3 | Caucasian | Steatosis | Cardiac: Ischaemic heart disease (CAD) and upper gastrointestinal haemorrhage (UGIH) | No |
| **9** | 56 | 15 | 6.66 | NA | 7.8 | Caucasian | Steatosis | Cardiac: Ischaemic heart disease (CAD) and emphysema | No |
| **10** | 81 | 36 | 6.44 | Ex-smoker | 8.7 | Caucasian | Cirrhosis | Infection: Sepsis/ pneumonia | No |
| **11** | 50 | 17 | 6.3 | NA | 6.4 | Caucasian | Steatosis | Cardiac: Ischaemic heart disease | No |
| **12** | 50 | 24 | 6.59 | Yes | 8.4 | Caucasian | Cirrhosis | Blood loss | No |
| **13** | 52 | 45.5 | 6.78 | Yes | 5.8 | Caucasian | Steatosis | Respiratory: Lobar pneumonia | No |
| **14** | 53 | 60 | 6.75 | Yes | 7.6 | Caucasian | Congestion | Respiratory: Chronic airflow limitation | No |
| **15** | 54 | 17 | 6.41 | Yes | 8.3 | Caucasian | Steatosis | Trauma: Chest and abdominal injury, Ishaemic Heart Disease | No |
| **16** | 55 | 17 | 6.85 | No | 8.5 | Caucasian | Congestion | Respiratory | Depression for 17 years, no antidepressants |
| **17** | 55 | 48 | 7.02 | Yes | 8.6 | Caucasian | Cirrhosis | Cardiac | Depression/anxiety -6 years and treated with antidepressants |
| **18** | 56 | 65 | 6.47 | Yes | 5.9 | Caucasian | Cirrhosis | Cardiac | No |
| **19** | 58 | 44.5 | 6.47 | Yes | 8.2 | Caucasian | Cirrhosis | Cardiac | No |
| **20** | 58 | 20 | 6.64 | Yes | 6.8 | Caucasian | Cirrhosis | Cardiac | No |
| **21** | 60 | 51 | 6.7 | No | 7.3 | Caucasian | Cirrhosis | Hepatic | Depression. Several O/d's – overdosed on panadeine & dilantin. Last known meds were Aldactone 100 mg. 2/day; Lasix 40 mg/day & Campral.no antidepressant medication noted on files |
| **22** | 60 | 16.5 | 6.48 | Yes | 8.6 | Caucasian | Cirrhosis | Hepatic | No |
| **23** | 61 | 27.5 | 5.87 | Yes | 5.3 | Caucasian | Cirrhosis | Cardiac | Severe depression, history of depression over 7-10 years |
| **24** | 63 | 25.5 | 6.21 | Yes | 5.0 | Caucasian | Congestion | Cardiac | No |
| **25** | 64 | 39 | 6.76 | Yes | 9.0 | Caucasian | Steatosis | Toxicity | Depression 3 years treated with antidepressants. |
| **26** | 69 | 22 | 5.82 | Yes | 5.7 | Caucasian | Congestion | Toxicity | Depression 50 years, treated for 4 years ( 27 years prior) with antidepressants |
| **27** | 70 | 62 | 6.82 | Yes | 7.1 | Caucasian | Congestion | Cardiac: Cardiomyopathy | Depression and suicidal 12 months prior to death -treated with antidepressants |
| **28** | 70 | 32 | 6.05 | NA | 6.7 | Caucasian | Cirrhosis | Infection | No |
| **29** | 43 | 29 | 6.29 | Yes | 6.2 | Caucasian | Steatosis | Blood loss | No |

PMI, post-mortem interval; NA, not available; No, no history of psychiatric illness noted.

**Table S3.** Primers list for RT-qPCR

| **Genes** | **FD Primer Sequence** | **RV Primer Sequence** | **Amplicon Size (bp)** |
| --- | --- | --- | --- |
| GRIA1 (GluA1) | ACTGGAAGAGACCCAAGTACACCTC | AGACAATCCCCAGCATTCCCCC | 127 |
| GRIA2 (GluA2) | CGCAGTCACTAATGCTTTCTGCTCC | AGGAGACGTGGAGTGTTCCGCA | 119 |
| GRIA3 (GluA3) | ACACCATCAGCATAGGTGGA | CTTCTCGGTGGTGTTCTGGT | 107 |
| GRIA4 (Glu42) | GGACACTCAAACAGGTTCG | TTGTGTAATTGACTCTACGTCC | 80 |
| GRIK1 (GluK1) | AGCAACAAAGACAAGTCCA | GGGTTCTTCCAGAATGGTG | 81 |
| GRIK2 (GluK2) | AAAGTGGTCGATGGAACGA | TAGAGCAGCATCAGTCGTC | 85 |
| GRIK3 (GluK3) | AGAGAGCAGCGTTCCTTCTG | CGGCGGTCATTGAA TGTGT | 146 |
| GRIK4 (GluK4) | TGAGGATCGCTGCTATCTTGG | CGTACTCGCTGTCTCTGAGAA | 159 |
| GRIK5 (GluK5) | CAGGTGCTCTCATCACTGCG | CTGACACATGGTGTCCGTGGT | 198 |
| GRIN1 (GluN1) | CATCCTCAAGTCCCACGAG | TTCCTGATACCGAACCCAC | 67 |
| GRIN2A (GluN2A) | GAATGATCGGTGAAGTGGTC | CCACTTCAGAACGTTCCTC | 81 |
| GRIN2B (GluN2B) | GGCAGATAAGGATGAATCCTC | ATGATGTTGAGCATTACGGA | 81 |
| GRIN2C (GluN2C) | CTTCTTGGAGCCATATAGCC | AACATGAAGACGGTGATGG | 85 |
| GRIN2D (GluN2D) | ATGGTTTCCTTCCTGAGCT | ATGAAGTACCTATGCAGACTCTC | 82 |
| GRIN3A (GluN3A) | GCCATAGAAGGATACGGCA | CTTGTATTGACTGATTAGCTCGG | 81 |
| GRIN3B (GluN3B) | CCTGTCCGAGTTCATCAG | CTGCAGGGTCTCTGTAAC | 118 |
| GRID1 (GluD1) | TGGTGACTGTCTTGGAAGAG | TGGAGAACCCTTTGTAGCG | 81 |
| GRID2 (GluD2) | TTATCCTAAGAGTGGTCACAGAG | GTATTCCACGGATATCGTATTCAC | 81 |
| GABRA1 (α1) | GTCACCAGTTTCGGACCCG | AACCGGAGGACTGTCATAGGT | 119 |
| GABRA2 (α2) | GTTCAAGCTGAATGCCCAAT | ACCTAGAGCCATCAGGAGCA | 160 |
| GABRA3 (α3) | CAACTTGTTTCAGTTCATTCATCCTT | CTTGTTTGTGTGATTATCATCTTCTTAGG | 102 |
| GABRA4 (α4) | TTGGGGGTCCTGTTACAGAAG | TCTGCCTGAAGAACACATCCA | 105 |
| GABRA5 (α5) | TTGGATGGCTACGACAACAGA | GTCCTCACCTGAGTGATGCG | 62 |
| GABRA6 (α6) | ACCCACAGTGACAATATCAAAAGC | GGAGTCAGGATGCAAAACAATCT | 67 |
| GABRB1 (β1) | TGCATGTATGATGGATCTTCG | GTGGTATAGCCATAACTTTCGA | 80 |
| GABRB2 (β2) | GCAGAGTGTCAATGACCCTAGT | TGGCAATGTCAATGTTCATCCC | 137 |
| GABRB3 (β3) | CAAGCTGTTGAAAGGCTACGA | ACTTCGGAAACCATGTCGATG | 108 |
| GABRD (δ) | CTTTGCTCATTTCAACGCC | TTCCTCACGTCCATCTCTG | 86 |
| GABRE (ε) | ACAGGAGTGAGCAACAAAACTG | TGAAAGGCAACATAGCCAAA | 107 |
| GABRG1 (γ1) | CCTTTTCTTCTGCGGAGTCAA | CATCTGCCTTATCAACACAGTTTCC | 91 |
| GABRG2 (γ2) | CACAGAAAATGACGGTGTGG | TCACCCTCAGGAACTTTTGG | 136 |
| GABRG3 (γ3) | AACCAACCACCACGAAGAAGA | CCTCATGTCCAGGAGGGAAT | 113 |
| GABRP (π) | CAATTTTGGTGGAGAACCCG | GCTGTCGGAGGTATATGGTG | 110 |
| GABRR1 (ρ1) | Hs00266687_m1 from Applied Biosystem |  | 94 |
| GABRR2 (ρ2) | TACAGCATGAGGATTACGGT | CAAAGAACAGGTCTGGGAG | 81 |
| GABRR3 (ρ3) | TGATGCTTTCATGGGTTTCA | CGCTCACAGCAGTGATGATT | 111 |
| GABRQ (θ) | CCAGGGTGACAATTGGCTTAA | CCCGCAGATGTGAGTCGAT | 63 |
| ACTB (β-actin) | CCTGGCACCCAGCACAAT | GGGCCGGACTCGTCATACT | 144 |
| TBP | GAGCTGTGATGTGAAGTTTCC | TCTGGGTTTGATCATTCTGTAG | 117 |
| GAPDH | GTGAAGGTCGGAGTCAACGG | TCAATGAAGGGGTCATTGATGG | 107 |
| RPLP0 | CCTCATATCCGGGGGAATGTG | GCAGCAGCTGGCACCTTATTG | 95 |

**Table S4** Analysis of normality of RT-qPCR data distribution by Shapiro–Wilk normality test

|  | **Caudate** | **Putamen** | **Putamen - Caudate** |
| --- | --- | --- | --- |
| GluA1 | = 0.34 | < 0.05 | = 0.075 |
| GluA2 | < 0.05 | < 0.05 | < 0.05 |
| GluA3 | < 0.05 | < 0.05 | < 0.05 |
| GluA4 | < 0.05 | < 0.05 | < 0.05 |
| GluK1 | < 0.05 | < 0.05 | < 0.05 |
| GluK2 | = 0.076 | < 0.05 | < 0.05 |
| GluK3 | < 0.05 | < 0.05 | < 0.05 |
| GluK4 | = 0.19 | < 0.05 | < 0.05 |
| GluK5 | < 0.05 | < 0.05 | < 0.05 |
| GluN1 | < 0.05 | < 0.05 | < 0.05 |
| GluN2A | < 0.05 | < 0.05 | < 0.05 |
| GluN2B | < 0.05 | < 0.05 | < 0.05 |
| GluN2C | = 0.13 | < 0.05 | < 0.05 |
| GluN2D | < 0.05 | < 0.05 | < 0.05 |
| GluN3A | < 0.05 | < 0.05 | < 0.05 |
| GluN3B | < 0.05 | < 0.05 | < 0.05 |
| GluD1 | < 0.05 | < 0.05 | < 0.05 |
| GluD2 | < 0.05 | < 0.05 | < 0.05 |
| α1 | = 0.4 | = 0.21 | < 0.05 |
| α2 | = 0.42 | = 0.61 | = 0.61 |
| α3 | < 0.05 | < 0.05 | < 0.05 |
| α4 | = 0.7 | < 0.05 | = 0.9 |
| α5 | < 0.05 | < 0.05 | = 0.2 |
| α6 | < 0.05 | = 0.43 | = 0.82 |
| β1 | = 0.17 | = 0.14 | = 0.15 |
| β2 | = 0.98 | = 0.16 | = 0.87 |
| β3 | = 0.97 | = 0.57 | = 0.58 |
| γ1 | = 0.64 | = 0.22 | = 0.85 |
| γ2 | < 0.05 | < 0.05 | < 0.05 |
| γ3 | < 0.05 | < 0.05 | < 0.05 |
| δ | = 0.12 | < 0.05 | < 0.05 |
| ε | = 0.51 | < 0.05 | < 0.05 |
| θ | < 0.05 | = 0.21 | = 0.34 |
| π | < 0.05 | < 0.05 | < 0.05 |
| ρ1 | < 0.05 | < 0.05 | < 0.05 |
| ρ2 | < 0.05 | < 0.05 | < 0.05 |
| ρ3 | < 0.05 | < 0.05 | < 0.05 |

p<0.05 indicates the data are not normally distributed

**Table S5**. Subunits with low expression frequency in either caudate or putamen.

GluN3B, π, ρ1 and ρ2 subunits were not compared statistically due to their frequency of expression in the samples was low and varied between 17-54%. We statistically compared subunits that were expressed in equal to or more than 84.5 % of the samples.

| Subunit | Caudate | Putamen |
| --- | --- | --- |
| GluN3B | 48/58 = 82.76% | 11/58 = 19% |
| π | 10/58 = 17.24% | 25/58 = 43.1% |
| ρ1 | 20/58 = 34.48% | 31/58 = 53.45% |
| ρ3 | 18/58 = 31.04% | 14/58 = 24.14% |
| GluN3B in controls | 22/29 = 75.86% | 8/29 = 27.59% |
