# Supplementary material for: GABA-A and NMDA receptor subunit mRNA expression is altered in the caudate but not the putamen of the postmortem brains of alcoholics
Source: Front Cell Neurosci. 2014 Dec 5;8:415. doi: 10.3389/fncel.2014.00415 (PMC4257153; doi:10.3389/fncel.2014.00415)
Supplement: Supplementary file 2 [file DataSheet1.DOCX]

**
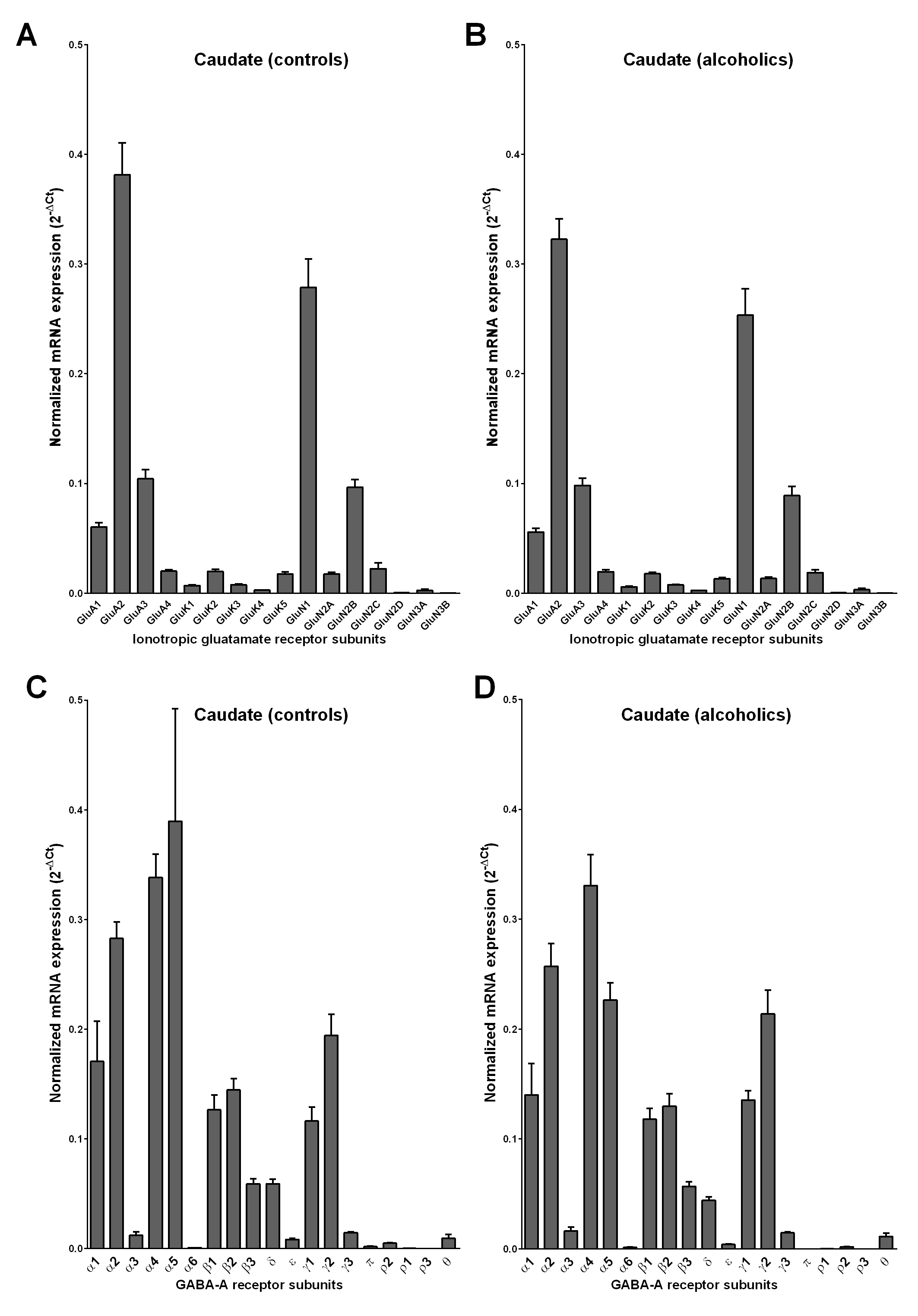
FIGURE S1: Expression of ionotropic glutamate and GABA-A receptor subunit mRNAs in the caudate of control (*n*** =29**) or alcoholic (*n*** =29**) subjects.** The mRNA level of each subunit was normalized to Act-B + GAPDH for (A) and (B) or Act-B + RPLP0 for (C) and (D) and presented as mean ± SEM.

**
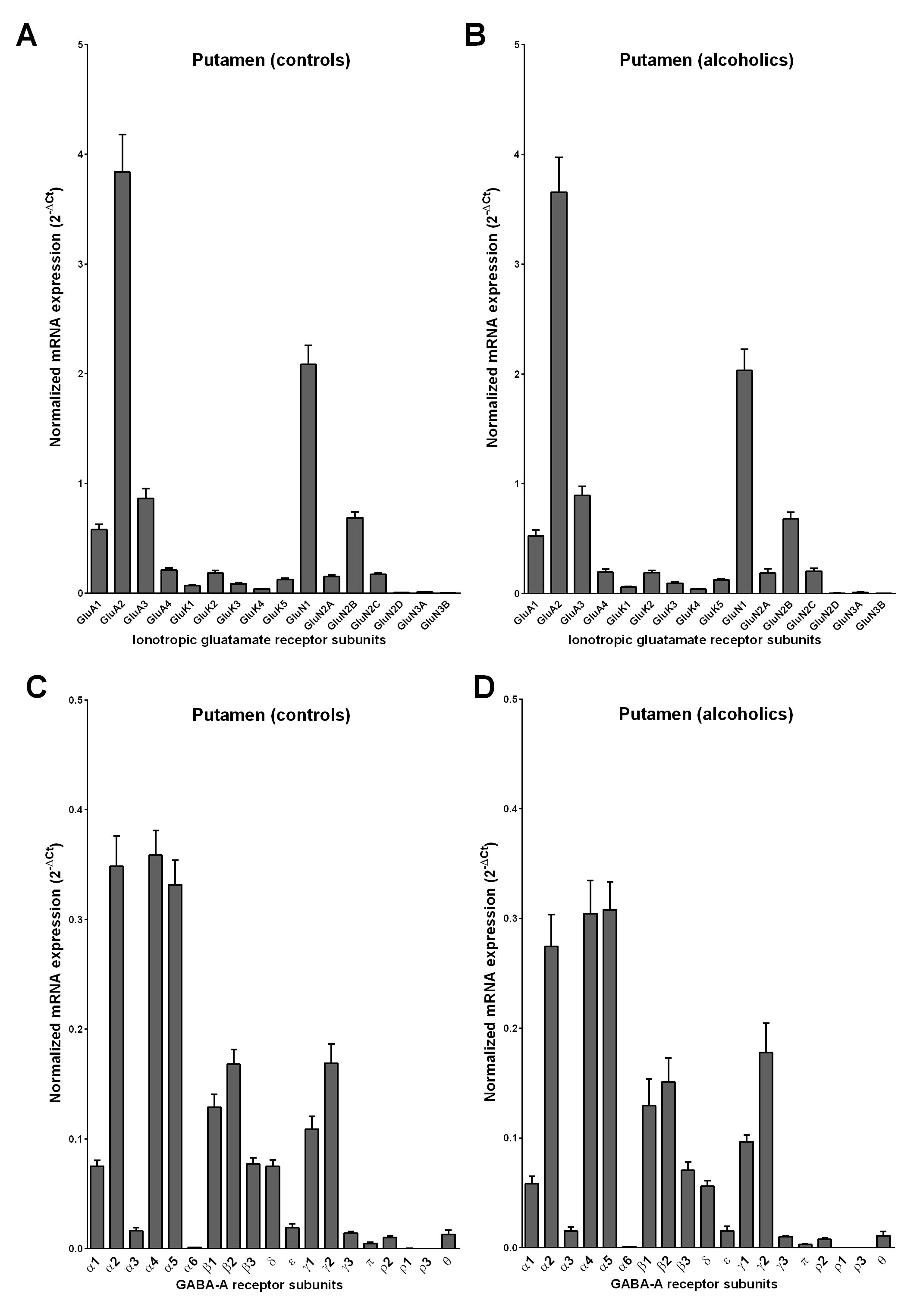
FIGURE S2: Expression of ionotropic glutamate and GABA-A receptor subunit mRNAs in the putamen of control (*n*** =29**) or alcoholic (*n*** =29**) subjects.** The mRNA level of each subunit was normalized to Act-B + TBP for (A) and (B) or Act-B + RPLP0 for (C) and (D) and presented as mean ± SEM.

**
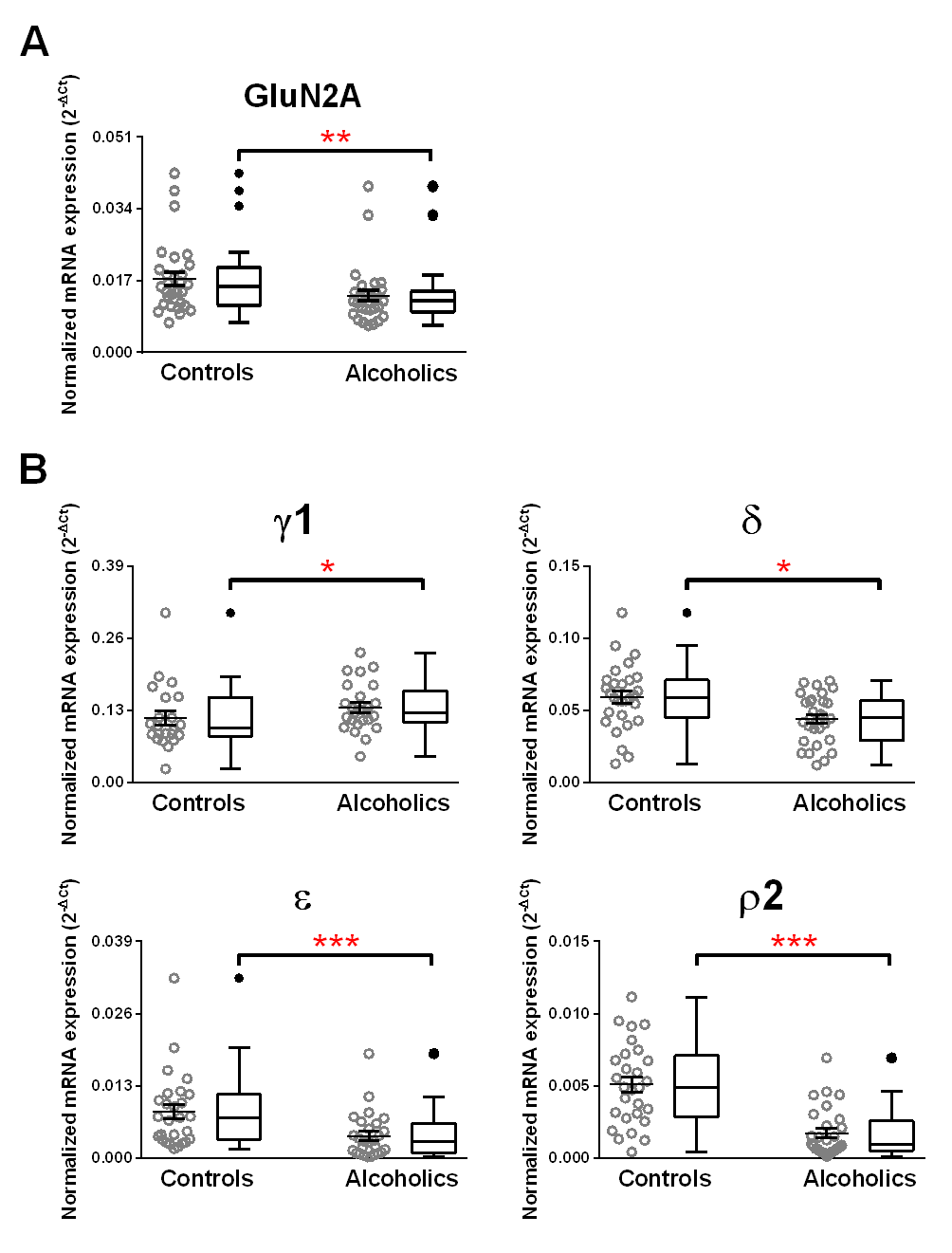
**

**FIGURE S3: Alcoholic subjects differed in specific ionotropic glutamate and GABA-A receptor subunits mRNA in the caudate of controls (*n*** =29**) and alcoholics (*n*** =29**).** The mRNA level of each subunit was normalized to Act-B + GAPDH for (A) and Act-B + RPLP0 for (B) and presented as scatter dot plot (◦) with mean and 95% confidence interval and box and whiskers plot with median and whiskers plotted by Tukey method to determine outliers (• - above or below the whiskers). Statistical analysis was performed by excluding outliers. One way ANOVA with Bonferroni *post hoc* test, GluN2A, df=51, *p* =0.0042; γ1, df=51, *p* =0.033; δ, df=51, *p* =0.011; Kruskal–Wallis ANOVA on ranks with Dunn’s *post hoc* test, ε, *H*(1, 52)=11.22, *p* =0.0008; ρ2, *H*(1, 55)=23.51, *p* =0.0000.
